# KAT6A chimeras form a self-reinforcing epigenetic module with NURF and MLL/COMPASS to sustain AML

**DOI:** 10.1186/s13059-025-03743-y

**Published:** 2025-08-19

**Authors:** Junhui Lv, Zhinang Yin, Conghui Li, Honglin Wen, Jian Ni, Peiyuan Yang, Zemin Song, Ying Xiang, Honghong Wang, Rui Lu, Li Huang, Ying Zhou, Hai-Bing Zhou, Ruijing Xiao, Pingping Fang, Kaiwei Liang

**Affiliations:** 1 State Key Laboratory of Metabolism and Regulation in Complex Organisms, TaiKang Center for Life and Medical Sciences, School of Basic Medical Sciences, Wuhan University, 115 DongHu Road, Research Building III, Room 404, Wuchang District, Wuhan, 430071 China; 2 State Key Laboratory of Virology and Biosafety, Frontier Science Center for Immunology and Metabolism, School of Pharmaceutical Sciences, Wuhan University, Wuhan, 430071 China; 3 Research Center for Medicine and Structural Biology, School of Basic Medical Sciences, Wuhan University, Wuhan, China; 4 Hubei Province Key Laboratory of Allergy and Immunology, Department of Pathophysiology, School of Basic Medical Sciences, Wuhan University, Wuhan, China

**Keywords:** Acute myeloid leukemia, AML, KAT6A, KAT6A-CBP, KAT6A-P300, Nucleosome Remodeling Factor, NURF, MLL/COMPASS, Chromatin accessibility

## Abstract

**Background:**

KAT6A-CBP (K/C) and KAT6A-P300 (K/P) fusions are recurrent genetic alterations in acute myeloid leukemia (AML) associated with poor prognosis. Despite their strong oncogenic potential, the mechanisms underlying their genomic targeting and leukemogenic function remain unclear. A major challenge has been their large size, which has impeded preclinical model development and mechanistic studies.

**Results:**

We employ a domain-focused truncation strategy to generate de novo murine models of K/C and K/P fusions, which faithfully recapitulate the morphological, immunophenotypic, and transcriptomic features of KAT6A-rearranged AML. Genomic profiling reveals that KAT6A fusions preferentially localize to H3K4me2/3-marked regions, while biochemical analyses uncover that KAT6A interacts with the Nucleosome Remodeling Factor (NURF), a key H3K4me2/3 reader. Disrupting NURF-chromatin interactions via depletion or small-molecule inhibition of its subunit, Bromodomain PHD Finger Transcription Factor (BPTF), impairs K/C recruitment and disrupts MLL/COMPASS-mediated H3K4me2 deposition, defining a functional epigenetic module involving KAT6A chimeras, NURF, and MLL/COMPASS. Notably, CBP/P300 inhibition reduces histone acetylation and chromatin accessibility, further impairing the recruitment of this epigenetic module. Targeting this module via NURF or CBP/P300 inhibition demonstrates efficacy in K/C leukemia models, with enhanced therapeutic effects observed when combined.

**Conclusions:**

Our study identifies a self-reinforcing epigenetic module of histone modifiers and readers in KAT6A-rearranged AML, providing mechanistic insights into the genomic targeting of KAT6A chimeras and highlighting promising combinatorial therapeutic strategies.

**Supplementary Information:**

The online version contains supplementary material available at 10.1186/s13059-025-03743-y.

## Background

Acute myeloid leukemia (AML) is the most common leukemia in adults, yet long-term survival rates remain dismally low despite significant therapeutic advancements [1, 2]. A defining feature of AML is the frequent occurrence of chromosomal translocations that produce oncogenic fusion proteins, driving leukemogenesis from hematopoietic stem and progenitor cells (HSPCs) [3–5]. Among these, Lysine Acetyltransferase 6 A (KAT6A, also known as MOZ) fusions are particularly prominent [6–8]. KAT6A, a member of the MYST family of histone acetyltransferases, is a critical regulator of HOX gene expression, essential for embryogenesis and hematopoiesis [9–11]. It achieves this through interactions with chromatin regulators, including bromodomain-PHD finger protein 1 (BRPF1), inhibitor of growth (ING) proteins, MYST/Esa1-associated factor 6 (MEAF6), as well as through collaboration with the H3K4 methyltransferase MLL/COMPASS [12–14].

In AML, recurrent chromosomal rearrangements fuse KAT6A to transcriptional coactivators, including P300 (EP300), CBP (CREBBP), and nuclear receptor coactivators (NCOA3 and TIF2). These fusions are associated with distinct clinical features such as monocytic or myelomonocytic differentiation, leukemia cutis, erythrophagocytosis, as well as high-risk outcomes including disseminated intravascular coagulation and poor survival [7, 15]. CBP and P300, paralogous histone acetyltransferases with conserved domain architectures, serve as transcriptional coactivators by bridging chromatin remodelers and transcription factors. Despite their critical oncogenic role, KAT6A translocations are rare (< 1%), with the most frequent subtype, KAT6A-CBP(K/C), occurring in only 0.2–0.4% of AML cases [16, 17]. CBP and P300 bridge transcription factors to the general transcription machinery and promote chromatin relaxation through intrinsic acetyltransferase activity [18, 19]. Conserved domains, such as the cysteine-histidine-rich (C/H) regions and functional modules like TAZ2, KIX, bromodomain, Q/P-rich region, and HAT domain, may mediate the oncogenic functions of KAT6A fusions [20]. However, whether these domains are truly essential for leukemogenesis remains unknown, and further investigation is needed to determine their specific contributions to the pathogenesis of KAT6A-rearranged AML.

Despite their potent oncogenic activity, the mechanisms by which KAT6A fusion proteins achieve genomic specificity and drive leukemogenesis remain poorly understood [21]. Previous studies suggest that the leukemogenic potential of KAT6A-TIF2 depends on its chromatin-binding ability rather than its histone acetyltransferase (HAT) activity, with oncogenicity further amplified by CBP recruitment through its fusion partners [22, 23]. The winged helix domain (WH1) of KAT6A can bind unmethylated CpG islands and interact with P300/CBP via the TAZ2 domain [24–26]. However, the widespread presence of unmethylated CpG islands across the genome [27] does not fully explain the genomic specificity of KAT6A-P300 (K/P) and K/C fusions, leaving key mechanistic gaps unexplored.

Adding to these challenges is the size of CBP and P300, each exceeding 2400 amino acids and forming fusion proteins of approximately 3500 amino acids when fused with KAT6A. These large proteins pose significant technical barriers for traditional retroviral expression systems due to their limited packaging capacity, hampering efficient expression and robust in vivo modeling [28, 29]. To overcome these limitations, we employed a domain-focused truncation strategy to generate functional K/C and K/P fusion proteins. These truncated constructs, preserving the essential N-terminal domain of KAT6A and critical CBP/P300 domains, facilitated the development of robust murine AML models. These models faithfully recapitulate the morphological, transcriptomic, and immunophenotypic features of KAT6A-rearranged AML. Leveraging these systems, we uncovered a critical epigenetic transcriptional module comprising KAT6A fusions, the Nucleosome Remodeling Factor (NURF) complex, and MLL/COMPASS. This self-reinforcing module sustains oncogenic transcriptional activation and drives AML progression. Notably, our findings identify this module as a promising therapeutic target in these rare but aggressive KAT6A-rearranged AML.

## Results

### Development of K/C and K/P Murine Leukemia Models Recapitulating Primary AML Morphology

**Fig. 1:**
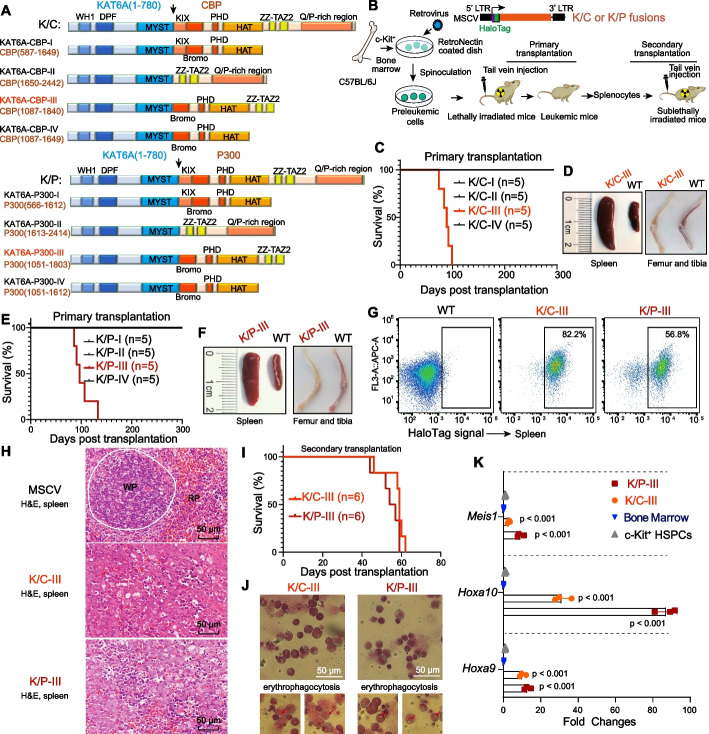
Establishment of de novo K/C and K/P murine leukemia models using a domain-focused truncation strategy. **A** Design of KAT6A-CBP (K/C) and KAT6A-P300 (K/P) truncations featuring different domains to circumvent the retroviral cargo limit. DPF: double PHD finger; WH1: winged helix (WH) domains. **B** Schematic overview of a retrovirus-based murine leukemia model derived from c-Kit-enriched HSPCs, utilizing K/C or K/P truncations to induce leukemia in mice. **C**, **D** Kaplan–Meier survival curves for C57BL/6J mice transplanted with HSPCs transduced with K/C constructs, assessing oncogenic potential (*n* = 5 mice/group). Endpoint examinations of leukemic mice revealed splenomegaly and pale bone marrow in both the femur and tibia. **E**, **F** Kaplan–Meier survival analysis of C57BL/6J mice transplanted with HSPCs expressing K/P truncations (*n* = 5 mice/group). Endpoint analysis confirmed leukemic characteristics in the leukemic cohort. **G** Flow cytometry analysis of HaloTag signals confirmed extensive infiltration of leukemic blasts in the spleens of leukemic mice. **H** H&E-stained spleen sections from the indicated cohorts, shown at 20 × magnification. The white pulp (WP) is outlined in white for samples from mice transplanted with MSCV empty vector-infected HSPCs (top). Notably, the clear demarcation between white pulp and red pulp (RP) is lost in mice receiving K/C-III and K/P-III due to excessive expansion of transformed leukemia cells, resulting in the splenomegaly observed in Fig. 1D and 1F. **I** Secondary transplantation study using primary leukemic spleen cells from mice initially transduced with K/C-III or K/P-III, assessing leukemogenic capacity (*n* = 6). **J** Wright-Giemsa staining of leukemic cells revealed both mature and immature myeloid cells, many with monocytic features, alongside some blast forms. Erythrophagocytosis was observed in both K/C-III and K/P-III leukemic cells. **K** RT-qPCR analysis of Meis1, Hoxa9, and Hoxa10 gene expression in c-Kit-enriched HSPCs, normal bone marrow cells, and K/C-III and K/P-III leukemic cells. Both K/C-III and K/P-III leukemic cells exhibited elevated expression of these known KAT6A chimera target genes. One-way ANOVA was used for statistical analysis

To address the challenges posed by large sizes of full-length K/C and K/P fusion proteins (~ 3500 amino acids), we employed a domain-focused truncation strategy to generate four truncated KAT6A fusion variants incorporating key functional domains of CBP or P300 (Fig. 1A). Specifically, K/C-I included the KIX, bromodomain, PHD, and HAT domains; K/C-II comprised the ZZ-TAZ2 and Q/P-rich regions; K/C-III incorporated the bromodomain, PHD, HAT, and ZZ-TAZ2 domains; while K/C-IV omitted the ZZ-TAZ2 domain from K/C-III (Fig. 1A). Using a optimized retrovirus-based system with HaloTag tracking in our lab [30], we transduced c-Kit-enriched murine HSPCs and enhanced transduction efficiency via RetroNectin-coated plates with spinoculation (Fig. 1B; Additional file 1: Fig. S1A). Transduced cells were transplanted into lethally irradiated C57BL/6 mice for leukemia development monitoring.

Strikingly, all mice (5/5) transplanted with K/C-III-transduced cells developed AML within 100 days post-transplantation, whereas mice receiving other K/C variants or controls remained disease-free (Fig. 1C; Additional file 1: Fig. S1B and C). Leukemic mice displayed hallmark AML features, including splenomegaly and pale, leukemic bone marrow in femurs and tibiae (Fig. 1D). Consistent results were observed with K/P truncation constructs (Fig. 1A and E). Mice expressing K/P-III developed classic AML features, such as pale bone marrow, splenomegaly, and infiltration of HaloTag-positive leukemic blasts (Fig. 1F and G). These findings underscore the critical roles of the HAT and ZZ-TAZ2 domains of CBP/P300 in leukemogenesis, likely attributable to their histone-binding capacity and regulation of HAT specificity and activity of CBP/P300 [31, 32]. In contrast, the KIX domain and Q/P-rich region were found to be dispensable.

Histological examination revealed extensive leukemic infiltration and disrupted splenic architecture (Fig. 1H). Secondary transplantation experiments confirmed the aggressive and persistent nature of leukemia driven by K/C-III and K/P-III constructs (Fig. 1I). Giemsa staining of leukemic cells demonstrated differentiation at the myelomonocytic stage, along with erythrophagocytosis (Fig. 1J), closely resembling the morphology observed in human KAT6A-rearranged AML [7, 22]. Furthermore, transcriptomic analysis showed significant upregulation of key transcriptional targets of KAT6A fusions [8], including *Meis1*, *Hoxa9*, and *Hoxa10*, in K/C-III and K/P-III leukemic cells compared to myeloid progenitors (MPs, Lin^−^Sca1^−^c-Kit^+^) and normal bone marrow cells (Fig. 1K). These results underscore the fidelity of our K/C and K/P murine models in recapitulating the key features of primary KAT6A-rearranged AML.

### Immunotyping and Transcriptional Profiling of K/C and K/P Leukemic Cells

**Fig. 2:**
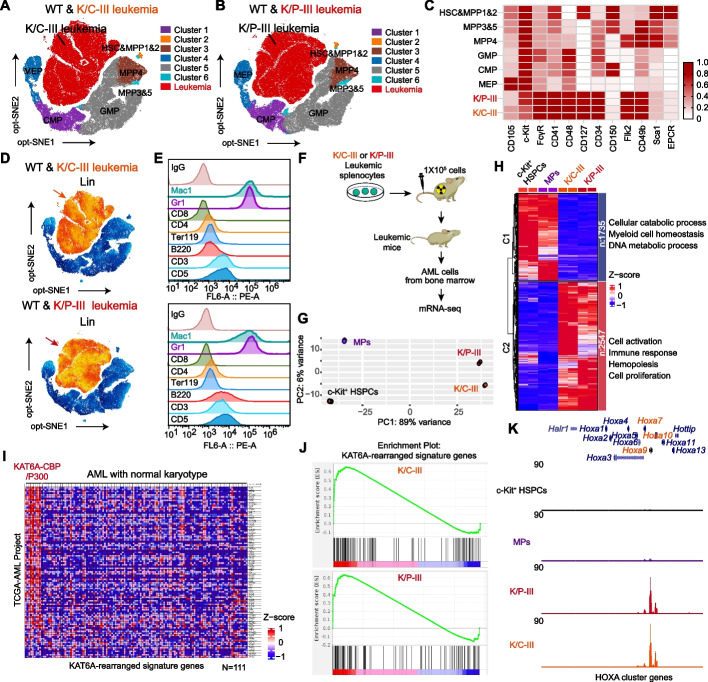
Immunotyping and transcriptome analysis of K/C and K/P murine leukemia cells. **A**, **B** Spectral flow cytometry of leukemia cells induced by K/C-III and K/P-III constructs. High-dimensional opt-SNE analysis, using all parameters except lineage and live/dead status, effectively distinguishes K/C-III and K/P-III AML cells (red) from normal murine hematopoietic cells. **C** Surface marker analysis of K/C-III and K/P-III AML cells, showing positive expression for CD34, FcγR, Flk2, CD41, CD48, and c-Kit (*n* = 2). **D** K/C-III and K/P-III AML cells exhibit lineage specification markers, including Ter119, Mac1, Gr1, B220, CD5, CD3, CD4, and CD8. The orange arrows indicate K/C-III and K/P-III leukemia cells. **E** Individual lineage marker analysis reveals strong expression of myeloid markers Mac1 and Gr1. **F** Workflow depicting isolation of K/C-III and K/P-III leukemia cells and subsequent mRNA sequencing. **G** Principal component analysis of mRNA-seq data from K/C-III and K/P-III leukemia cells, illustrating distinct transcriptional landscapes compared to MPs and c-Kit-enriched HSPCs (*n* = 2). **H** Heatmap of shared differentially expressed genes in K/C-III and K/P-III leukemia cells versus MPs, highlighting distinct expression profiles. Gene ontology analysis of these differentially expressed genes is also shown. C1 and C2 represent the downregulated and upregulated gene clusters, respectively, compared to MPs. **I** Enrichment analysis of the KAT6A-rearranged AML transcriptional signature in comparison to AML cases with normal karyotypes. RNA-seq data from the TARGET AML Project (TARGET-AML) includes cases with KAT6A rearrangements (*n* = 7) and normal karyotypes (*n* = 111). **J** GSEA analysis of K/C-III and K/P-III leukemia cells compared to MPs, showing enrichment of the KAT6A-rearranged signature genes. **K** RNA-seq tracks demonstrate upregulation of *Hoxa* cluster genes in K/C-III and K/P-III leukemia cells compared to c-Kit-enriched HSPCs and MPs

To characterize K/C and K/P leukemic cells, we conducted 15-fluorochrome spectral cytometry on bone marrow samples, using key markers to define the hematopoietic compartment architecture [33]. Phenotypic analysis revealed distinct immunoprofiles for K/C and K/P leukemia (Fig. 2A and B). Leukemic cells were distinguished from normal murine hematopoietic populations by markers such as CD34, FcγR, Flk2, and CD48, with subsets expressing c-Kit, CD49b, and CD41 (Fig. 2C; Additional file 1: Fig. S2A and B). Both subtypes were positive for lineage specification markers —a combination of Ter119, Mac1, Gr1, B220, CD5, CD3, CD4, and CD8 (Fig. 2D). Individual analysis of lineage markers showed predominant expression of myeloid markers Mac1 and Gr1 in the AML cells (Fig. 2E), which aligns with Giemsa staining that indicated differentiation at the myelomonocytic stage (Fig. 1J).

For transcriptional profiling, we performed mRNA sequencing and principal component analysis (PCA) on sorted leukemia cells, c-Kit-enriched HSPCs, and MPs. Leukemia cells displayed significant transcriptional divergence from normal HSPCs and MPs, with highly similar profiles between K/C-III and K/P-III variants (Fig. 2F and G). Differential expression analysis identified 1,735 downregulated and 2,547 upregulated genes in K/C-III and K/P-III leukemic cells compared to MPs (Fig. 2H; Additional file 2: Table S1). Gene ontology analysis of upregulated genes revealed enrichment in pathways related to cell activation, immune response, hematopoiesis, and cell proliferation, while downregulated genes were associated with cellular catabolism, myeloid homeostasis, and DNA metabolic processes (Fig. 2H).

As KAT6A-rearranged AML is characterized by a distinct transcriptional signature, including upregulation of the HOXA gene cluster [8], we analyzed the TARGET AML expression dataset. This confirmed a unique gene signature in KAT6A-rearranged AML cases (*n* = 7) compared to AML cases with normal karyotypes (*n* = 111) (Fig. 2I). Gene set enrichment analysis (GSEA) of murine K/C and K/P leukemia cells showed significant enrichment of the KAT6A-rearranged AML signature (Fig. 2J). Furthermore, tracks of individual genes verified robust upregulation of *Hoxa7*, *Hoxa9*, and *Hoxa10* in K/C-III and K/P-III leukemic cells compared to c-Kit -enriched HSPCs or MPs (Fig. 2K). These findings confirm that our K/C and K/P murine leukemia models faithfully replicate the immunophenotypic and transcriptional features of primary KAT6A-rearranged AML, providing robust systems for investigating disease mechanisms and therapeutic strategies.

### KAT6A Co-Localizes with H3K4me2/3 and Interacts with the NURF Complex

**Fig. 3:**
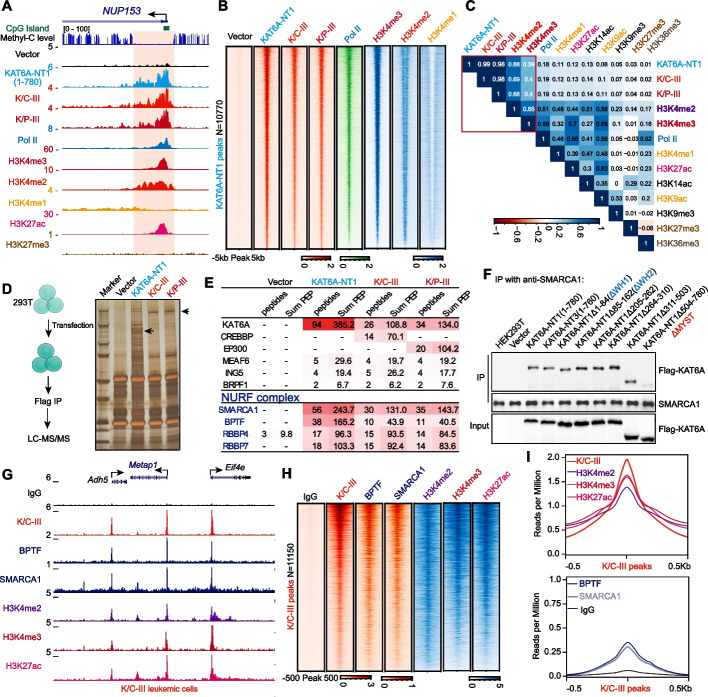
KAT6A associates with H3K4me2/3 and directly interact with the NURF complex. **A** Genome browser tracks showing the distribution of CpG islands, methyl-C levels, KAT6A-NT1, K/C-III, K/P-III, Pol II, and histone markers in HEK293T cells. CUT&RUN analyses of KAT6A-NT1, K/C-III, and K/P-III reveal similar binding profiles with occupancy extending beyond CpG islands, overlapping with H3K4me3 and H3K4me2. **B**, **C** Heatmap analysis of KAT6A-NT1, K/C-III, K/P-III, Pol II, and histone markers in HEK293T cells (B). KAT6A-NT1 peaks (*N* = 10,770) were ranked by decreasing occupancy, with Pearson correlation analysis (C) showing positive correlations between KAT6A-NT1, K/C-III, and K/P-III with H3K4me2 and H3K4me3 across the genome. **D** IP-MS analysis comparing KAT6A-NT1, K/C-III, and K/P-III to MSCV vector control in HEK293T cell lysates. Silver staining of IP products is displayed on the right. **E** Mass spectrometry analysis of KAT6A-NT1, K/C-III, and K/P-III immunoprecipitates. The peptide and sum PEP score for the BRPF1-KAT6A acetyltransferase complex and NURF complex subunits are presented. MSCV vector serves as the binding control. The list of interacting proteins was included in Additional file 2: Table S2. **F** Confirmation of the NURF complex interaction with KAT6A in HEK293T cells, demonstrated through anti-SMARCA1 immunoprecipitation followed by immunoblotting. KAT6A mutants were included, showing that deletion of the KAT6A MYST domain impairs the interaction with the NURF complex. **G** Colocalization of the NURF complex and K/C-III in murine K/C-III leukemia cells. Examples of genome browser tracks for BPTF, SMARCA1, HaloTag-K/C-III CUT&RUN, and ChIP-seq signals for H3K4me2, H3K4me3, and H3K27ac at the *Metap1* and *Adh5* loci are shown. **H** Genome-wide analysis demonstrating the colocalization of K/C-III with the NURF complex and H3K4me2 and H3K4me3. Heatmaps are sorted by decreasing K/C-III occupancy. **I** Metaplots of BPTF, SMARCA1, HaloTag-K/C-III, H3K4me2, H3K4me3, and H3K27ac at K/C-III peaks in murine leukemia cells

To investigate the genomic targeting of KAT6A chimeras, we performed CUT&RUN analysis on K/C-III, K/P-III, and the KAT6A N-terminus, examining their genome-wide binding patterns alongside Pol II and histone modifications, including H3K4me1/2/3, H3K36me3, H3K27me3, and H3K27ac. KAT6A N-terminus, K/C-III, and K/P-III proteins showed extensive promoter binding across the genome (Fig. 3A; Additional file 1: Fig. S3A), with distribution extending beyond CpG islands, indicating alternative mechanisms beyond WH1 domain-mediated recognition of unmethylated CpG islands [24, 25]. Heatmap and Pearson correlation analyses revealed a strong association with H3K4me2 and a moderate association with H3K4me3 (Fig. 3B and C). Similar patterns for KAT6A-TIF2 and KAT6A-NCOA3 chimeras supported the genome-wide colocalization of KAT6A with H3K4me2/3 (Additional file 1: Fig. S3B). Correlations between the KAT6A N-terminus and its chimeric variants demonstrated that the N-terminus dictates their genomic distribution (Additional file 1: Fig. S3C-F).

To uncover protein interactions underlying these genomic associations, we performed immunoprecipitation and mass spectrometry (IP-MS) on KAT6A N-terminus and chimeras (Fig. 3D). This analysis identified core components of the KAT6A complex, including BRPF1, ING5, and MEAF6 (Fig. 3E; Additional file 2: Table S2; Additional file 1: Fig. S3G). Notably, we noticed that KAT6A and its chimeras interacted with the NURF complex, comprising BPTF, SMARCA1, RBBP4, and RBBP7 (Fig. 3E; Additional file 1: Fig. S3G; Additional file 2: Table S2). Deletion mutant studies revealed that the MYST domain of KAT6A is essential for binding the NURF complex (Fig. 3F). Notably, the BPTF subunit of the NURF complex binds to H3K4me2/3 via its PHD finger domain [34, 35], likely explaining the observed colocalization of KAT6A with these histone markers.

To confirm the genomic associations among the NURF complex, KAT6A chimeras, and H3K4me2/3 in leukemia cells, we conducted CUT&RUN and ChIP-seq analyses in K/C-III leukemia cells. These analyses revealed significant colocalization of K/C-III with NURF components (BPTF and SMARCA1) and the histone modifications H3K4me2/3 (Fig. 3G and I). Scatter plot analyses further demonstrated strong positive correlations between K/C-III binding and NURF complex components (Additional file 1: Fig. S3H and I), confirming a genome-wide association of K/C-III with the NURF complex. Together, these findings suggest that KAT6A chimeras collaborate with the NURF complex, likely leveraging interactions with NURF to regulate leukemogenic gene expression programs.

### Disruption of NURF-Chromatin Interaction Impairs Genomic Targeting of KAT6A Chimeras

Given the interaction between the NURF complex and the MYST domain of KAT6A (Fig. 3F), as well as the NURF complex’s ability to bind histone modifications, we first examined how KAT6A mutants affect chromatin association. CUT&RUN analysis revealed that the KAT6A-ΔMYST mutant exhibited significantly reduced ~ 50% genome-wide binding compared to wild-type KAT6A, whereas a catalytically inactive mutant did not show this effect (Additional file 1: Fig. S4A-C). These findings indicate that the MYST domain, independent of its catalytic activity, is critical for KAT6A’s chromatin association.

**Fig. 4:**
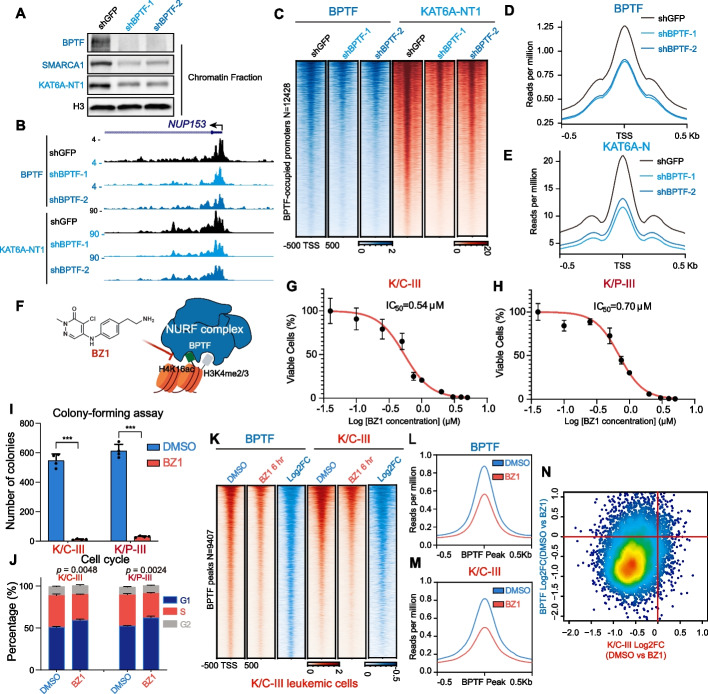
BPTF sustains the genome-wide targeting of KAT6A and KAT6A chimeras. **A** BPTF knockdown reduces KAT6A chromatin association. shRNA-mediated knockdown of BPTF in Flag-KAT6A-NT1-expressing HEK293T cells followed by chromatin fractionation and immunoblotting using anti-BPTF, anti-SMARCA1, and anti-Flag antibodies. **B** CUT&RUN analysis of BPTF and Flag-KAT6A occupancy after BPTF knockdown in Flag-KAT6A-NT1-expressing HEK293T cells. BPTF depletion decreases KAT6A occupancy at the *NUP153* locus. **C** Genome-wide analysis of BPTF-occupied promoters (*n* = 12,428) showing a global reduction in Flag-KAT6A occupancy following BPTF knockdown. Heatmaps are ranked by decreasing BPTF occupancy. **D**, **E** Metaplots of CUT&RUN signals for BPTF and Flag-KAT6A at TSS following BPTF knockdown. Depletion of BPTF reduces KAT6A occupancy across the genome. **F** Chemical structure of BZ1, a pyridazinone-based inhibitor targeting the BPTF bromodomain [36]. **G**, **H** Dose–response analysis of BZ1 in K/C-III and K/P-III leukemia cells, showing IC50 values. **I** BZ1 treatment inhibits colony formation in K/C-III and K/P-III leukemia cells. Leukemia cells were treated with 2 μM BZ1 for one week. **J** BZ1 induces G1 cell cycle arrest in K/C-III and K/P-III leukemia cells. Leukemia cells were treated with 2 μM BZ1 for 48 h (two-way ANOVA). **K**-**M** Genome-wide analysis of BPTF and K/C-III occupancy in murine K/C-III leukemia cells following 2 μM BZ1 treatment for 6 h. Heatmaps of BPTF peaks (*n* = 9,407) show reduced occupancy of BPTF and K/C-III. **N** Scatter plot illustrating log_2_ fold changes of BPTF and K/C-III signals at BPTF peaks after 6 h BZ1 treatment

Further investigation using BPTF knockdown demonstrated that BPTF depletion reduced KAT6A chromatin occupancy, as shown by chromatin fractionation and CUT&RUN analyses (Fig. 4A-C). Metaplot analyses revealed a ~ 50% reduction in KAT6A occupancy following BPTF knockdown (Fig. 4D and E). To disrupt BPTF-chromatin interaction, we used BZ1, a small-molecule inhibitor that blocks BPTF binding to H4K16ac [36] (Fig. 4F). BZ1 treatment resulted in dose-dependent inhibition of cell proliferation in K/C-III and K/P-III leukemia cells, with IC50 values of 0.54 µM and 0.70 µM, respectively (Fig. 4G and H). Additionally, BZ1 suppressed colony formation and induced G1 cell cycle arrest in both leukemia models, with minimal effects on cell differentiation (Fig. 4I and J; Additional file 1: Fig. S5A and B). In contrast, HSPCs exhibited an IC50 of 6.9 µM for BZ1—over tenfold higher than that observed in K/C-III and K/P-III leukemia cells (IC50 = 0.54–0.70 µM)—and showed minimal toxicity to normal hematopoietic progenitors (Additional file 1: Fig. S5C and D), indicating a favorable therapeutic window.

To generalize these findings, we tested BZ1 in murine models of KAT6A-rearranged leukemia, including KAT6A-TIF2 and KAT6A-NCOA3 (Additional file 1: Fig. S6). Consistent with observations in K/C-III and K/P-III models, BZ1 inhibited cell proliferation and colony formation, and induced G1 arrest in these leukemia cells as well (Additional file 1: Fig. S6I-L). A six-hour BZ1 treatment of K/C-III leukemia cells led to reduced genome-wide occupancy of both BPTF and K/C-III at BPTF-bound promoter regions (Fig. 4K-M). Log2 fold-change scatter plots of BPTF and K/C-III signals at BPTF peaks further demonstrated a reduction in their occupancy following BZ1 treatment (Fig. 4N). Collectively, these results highlight the critical role of the NURF complex, via BPTF, in mediating the genomic targeting of KAT6A chimeras in leukemia cells.

### NURF Sustains WDR5-MLL/COMPASS-deposited H3K4 Methylation

**Fig. 5:**
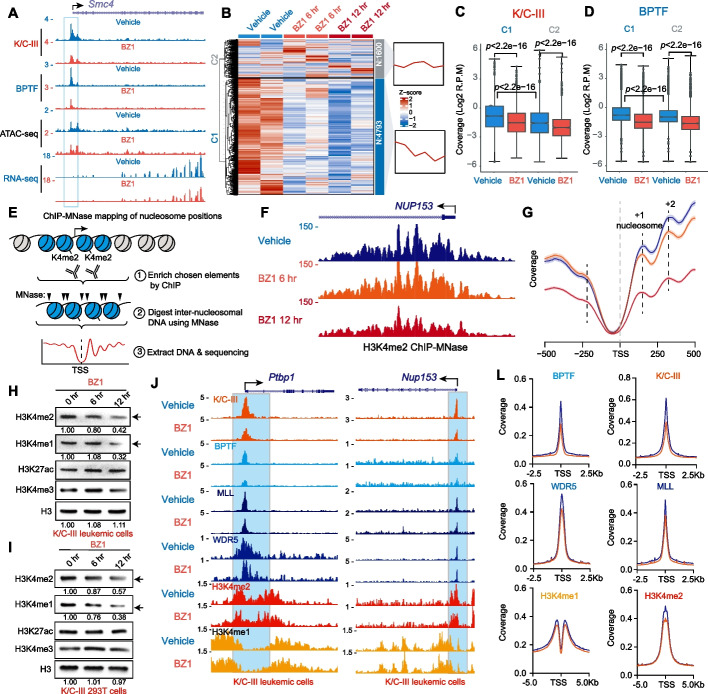
BPTF Inhibition diminishes H3K4 methylation and the chromatin association of WDR5-MLL/COMPASS. **A** CUT&RUN and RNA-seq tracks of BPTF and K/C-III in murine leukemia cells after 6 h of 2 μM BZ1 treatment, showing changes in chromatin occupancy. **B** RNA-seq analysis of K/C-III leukemia cells treated with 2 μM BZ1 for 6 or 12 h. K/C-III-bound genes are categorized into two clusters, with most genes in Cluster 1 exhibiting downregulation following BZ1 treatment. **C**, **D** Box plots showing K/C-III and BPTF signal intensities in K/C-III leukemia cells post-BZ1 treatment. Cluster 1 genes display higher K/C-III and BPTF signals compared to Cluster 2 genes. **E** Schematic of ChIP-MNase profiling of nucleosomes using an H3K4me2 antibody. **F** Example ChIP-MNase track at the *NUP153* locus, illustrating nucleosome positioning. HEK293T cells expressing K/C-III were treated with 2 μM BZ1 for 6 or 12 h. **G** ChIP-MNase mapping of nucleosome positions at promoter regions using an H3K4me2 antibody. Dashed lines indicate the positions of nucleosomes. **H**, **I** Immunoblot analysis showing a global reduction in H3K4me2 and H3K4me1 levels in murine K/C-III leukemia cells and K/C-III-expressing HEK293T cells by 2 μM BZ1 treatments for 6 or 12 h. **J**, **K** ChIP-seq and CUT&RUN analyses of K/C-III, BPTF, WDR5, MLL, and H3K4me1/2 in K/C-III leukemia cells after 6 h of BZ1 treatment. **L** Metaplot analysis of K/C-III, BPTF, WDR5, MLL, and H3K4me1/2 signals at TSS. BZ1 treatment reduces WDR5 and MLL occupancy, indicating disrupted chromatin association

To investigate the effects of BPTF inhibition on gene expression, we performed mRNA-seq on K/C-III leukemia cells following 6 and 12 h of BZ1 treatment (Fig. 5A; Additional file 2: Table S3). Gene ontology analysis of differentially expressed genes revealed enrichment in cell cycle-related pathways (Additional file 1: Fig. S7A), consistent with the observed G1 cell cycle arrest (Fig. 4J). Among K/C-III-bound genes, we identified two distinct clusters based on their response to BZ1. Cluster 1, which comprised the majority, exhibited a trend toward downregulation, whereas cluster 2 showed minimal changes in expression (Fig. 5B). Genes in cluster 2 had lower promoter occupancy by K/C-III and BPTF compared to cluster 1, suggesting that BZ1 preferentially affects genes with higher K/C-III and BPTF occupancy (Fig. 5C and D). Notably, chromatin accessibility remained largely unchanged at both cluster 1 and cluster 2 genes (Additional file 1: Fig. S7B and C).

As a chromatin remodeling complex, NURF facilitates ATP-dependent nucleosome sliding to regulate chromatin accessibility and transcription [37, 38]. BZ1 treatment reduced BPTF and K/C-III occupancy by ~ 50% (Fig. 4L and M). To assess the functional consequences of this reduction, we performed ChIP-MNase assays targeting H3K4me2-marked promoters colocalized with BPTF and K/C-III (Fig. 5E). Genome browser tracks revealed that BZ1 treatment significantly decreased H3K4me2 levels at the *NUP153* promoter (Fig. 5F). However, nucleosome profiling near transcription start sites (TSS) showed no significant nucleosome repositioning. Instead, it is essential for maintaining H3K4me2 methylation levels at promoters (Fig. 5G).

Further validation in K/C-III-expressing HEK293T cells and murine leukemia cells confirmed that BZ1 treatment reduced bulk levels of H3K4me1 and H3K4me2 (Fig. 5H and I). Given the established interactions between the NURF complex and the MLL/COMPASS complex component WDR5, as well as between KAT6A and the MLL complex [34, 39], we also confirmed that KAT6A and KAT6A chimeras interacts with WDR5 and RBBP5 in our IP-MS experiments (Additional file 2: Table S2). Next, we examined WDR5 and MLL occupancy at K/C-III-bound promoters using CUT&RUN (Fig. 5J and K). Following 6 h of BZ1 treatment, both WDR5 and MLL showed reduced binding, indicating that BPTF inhibition disrupts the recruitment of the WDR5-MLL/COMPASS complex, resulting in decreased H3K4me2 levels (Fig. 5L).

To determine whether the menin-MLL interaction is essential in KAT6A-rearranged leukemia, we evaluated the therapeutic potential of Revumenib, a clinical-stage menin-MLL inhibitor effective in MLL-rearranged AML [40]. KAT6A fusion-expressing leukemic cells treated with Revumenib (72 h) showed limited reduction in viability compared to control HSPCs (Additional file 1: Fig. S7D and E). Notably, high-dose Revumenib (6 μM) failed to induce cell cycle arrest (Additional file 1: Fig. S7F and G). These results suggest that MLL/COMPASS dependency in KAT6A-fusion leukemias may bypass the menin-MLL interaction, and underscores a distinct self-reinforcing mechanism whereby the NURF complex facilitates the recruitment of the WDR5-MLL/COMPASS complex to sustain H3K4 methylation. This epigenetic landscape likely enhances the occupancy of both NURF and KAT6A, supporting transcriptional regulation critical for leukemogenesis.

### CBP/P300 Inhibition Reduces Chromatin Accessibility and Recruitment of the KAT6A-NURF-MLL Module

**Fig. 6:**
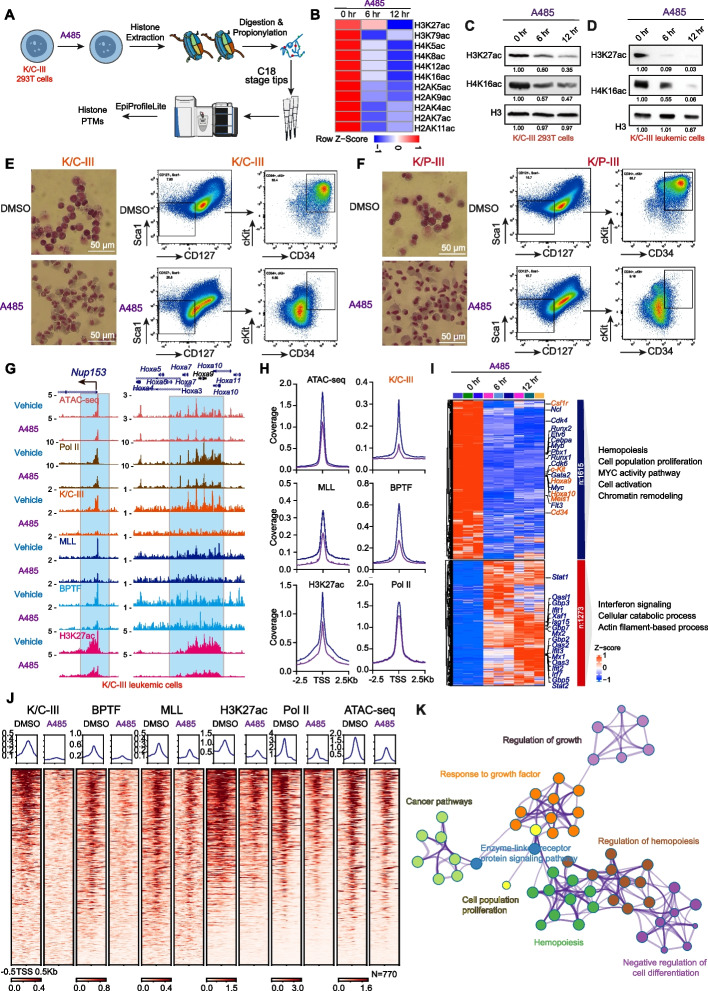
A485 disrupts histone acetylation and chromatin accessibility critical for the KAT6A-NURF-MLL module’s chromatin association. **A** Schematic illustrating the experimental workflow for profiling histone modifications in K/C-III 293 T cells following treatment of CBP/P300 acetyltransferase inhibitors A485 [19]. K/C-III 293 T cells were treated with 2 μM A485 for 6 or 12 h. **B** Heatmap showing a marked reduction in multiple histone acetylation marks in response to A485 treatments. **C**, **D** Immunoblot analysis confirms significant decreases in H3K27ac and H4K16ac levels in K/C-III 293 T cells and leukemia cells treated with 2μM A485 for 6 or 12 h. **E**, **F** A485 induces differentiation in K/C and K/P leukemia cells, as demonstrated by Wright-Giemsa staining and reduced expression of CD34 and c-Kit. **G** ATAC-seq, ChIP-seq, and CUT&RUN analyses of Pol II, K/C-III, BPTF, MLL, and H3K27ac in K/C-III leukemia cells after 6 h of 2 μM A485 treatment, showing reduced chromatin association at the *Nup153* and *Hoxa* cluster. **H** Metaplot analysis of ATAC-seq, Pol II, K/C-III, BPTF, MLL, and H3K27ac signals at promoters occupied by K/C-III. A485 treatment decreases chromatin accessibility, Pol II recruitment, and the chromatin association of the KAT6A-NURF-MLL module. **I** RNA-seq analysis of K/C-III leukemia cells treated with 2 μM A485 for 6 and 12 h. A485 downregulates genes involved in hematopoiesis, cell proliferation, and MYC activity pathways, while upregulating genes related to interferon signaling, cellular catabolic processes, and actin filament-based processes. **J** Identification of K/C core target genes. These 770 genes are bound by K/C-III and sensitive to A485 treatments. **K** GO analysis of these 770 core target genes revealed enrichment in leukemia stem cell maintenance, hemopoiesis, negative regulation of cell differentiation, and cell proliferation

To determine whether CBP/P300 HAT activity is essential for leukemia cell maintenance, we utilized the selective acetyltransferase inhibitor A485 [19]. Mass spectrometry analysis of post-translational modifications showed that A485 treatment of K/C-III-expressing HEK293T cells for 6 and 12 h rapidly reduced multiple histone acetylation marks, including H3K27ac, H3K79ac, H4K5ac, H4K8ac, H4K12ac, H4K16ac, H2AK5ac, H2AK9ac, H2AK4ac, H2AK7ac, and H2AK11ac (Fig. 6A and B). Notably, H4K16ac, a mark critical for interaction with BPTF to facilitate chromatin occupancy, was markedly affected. Immunoblotting confirmed decreases in H3K27ac and H4K16ac in K/C-III-expressing HEK293T cells and K/C-III leukemia cells upon A485 treatment (Fig. 6C and D).

Since A485 inhibits both endogenous CBP/P300 and the K/C fusion, we tested A485 in HEK293T isogenic cells to disentangle the contribution of K/C to A485. We found cells expressing K/C-III exhibited elevated H3K27ac levels compared to KAT6A-NT1 or MSCV controls. Short-term A485 treatment (6 h) preferentially reduced H3K27ac and H4K16ac marks in K/C-III cells (Additional file 1: Fig. S8A), suggesting rapid inhibition of fusion-dependent HAT activity. Dose–response assays further revealed a twofold lower IC50 for A485 in K/C-III cells (2.90 μM) compared to KAT6A-NT1 (5.37 μM) or MSCV controls (5.99 μM; Additional file 1: Fig. S8B), indicating heightened sensitivity to the A485 inhibitor. To validate these findings in primary HSPCs, we found K/C-III-expressing HSPCs showed twofold greater sensitivity to A485 in viability assays (IC50 = 2.37 μM vs. 6.0 μM for KAT6A-NT; Additional file 1: Fig. S8C). These results collectively suggest that K/C expression sensitizes cells to A485, likely due to the fusion’s dependence on CBP/P300 HAT activity. However, residual effects of A485 in control cells (e.g., MSCV, KAT6A-NT1) indicate that endogenous CBP/P300 inhibition may contribute to the phenotype at higher doses.

A485 treatment induced differentiation in leukemia cells, as evidenced by Giemsa staining, which revealed morphological changes starting from 4 days of treatment (Fig. 6E and F). Flow cytometry analysis showed reduced surface expression of c-Kit and CD34, markers of leukemic stemness, and similar differentiation effects were observed in KAT6A-TIF2 and KAT6A-NCOA3 leukemia cells, underscoring the broad impact of CBP/P300 inhibition (Additional file 1: Fig. S8D and E). To further explore the impact of CBP/P300 inhibition on chromatin dynamics, ATAC-seq was performed to assess chromatin accessibility [41]. Analysis of key factors, including Pol II, H3K27ac, BPTF, K/C, and MLL, in K/C-III leukemia cells revealed that A485 treatment for 6 h significantly decreased chromatin accessibility, Pol II loading, and the recruitment of the KAT6A-NURF-MLL module at *Nup153* and *Hoxa* cluster loci (Fig. 6G; Additional file 1: Fig. S8F). Genome-wide analyses confirmed similar reductions at all K/C-III-occupied promoter regions (Fig. 6H), indicating that CBP/P300 inhibition broadly disrupts chromatin accessibility and the recruitment of the KAT6A-NURF-MLL epigenetic module.

RNA-seq analysis of A485-treated K/C-III leukemia cells further revealed significant downregulation of key hematopoietic factors, including *Hoxa9*, *Hoxa10*, *Meis1*, *Cd34*, *c-Kit*, and *Csf1r* (Fig. 6I; Additional file 2: Table S4). To define the transcriptional networks directly regulated by KAT6A fusions, we integrated K/C-III CUT&RUN and RNA-seq datasets, and identified the core target genes of KAT6A fusion meeting two criteria: 1) Direct binding: occupied by KAT6A fusions in CUT&RUN data, 2) Transcriptional downregulation by A485. This integrative analysis identified 770 core target genes (Fig. 6J). K/C-III co-localizes with MLL and BPTF at these 770 core target genes (Fig. 6J), indicating cooperative interactions within the epigenetic module to amplify oncogenic transcription. GO analysis of these 770 core target genes revealed enrichment in leukemia stem cell maintenance (e.g., *Hoxa9, Meis1, Flt3*), hemopoiesis (e.g., *Runx1, Runx2, Cd34, Kit*), negative regulation of cell differentiation (e.g., *Gata2, Ldb1, Ptk2b*), and cell proliferation (e.g., *Ccnd1, Cdkn2c*) (Fig. 6K; Additional file 2: Table S5), which is consistent with oncogenic potential of KAT6A fusions.

Furthermore, to clarify why BZ1 causes cell cycle arrest but not differentiation (unlike A485), we analyzed the A485-downregulated genes and found that BPTF inhibition by BZ1 only slightly reduced occupancy of K/C-III, BPTF, and MLL, whereas A485 treatment resulted in a substantial reduction of these signals (Additional file 1: Fig. S8G and H). Similarly, BPTF inhibition via BZ1 did not significantly decrease chromatin accessibility or RNA Pol II occupancy at these loci, whereas A485 caused significant reductions (Additional file 1: Fig. S8G and H).

### Therapeutic Targeting of the KAT6A-NURF-MLL Module Delays K/C Leukemia Progression

**Fig. 7:**
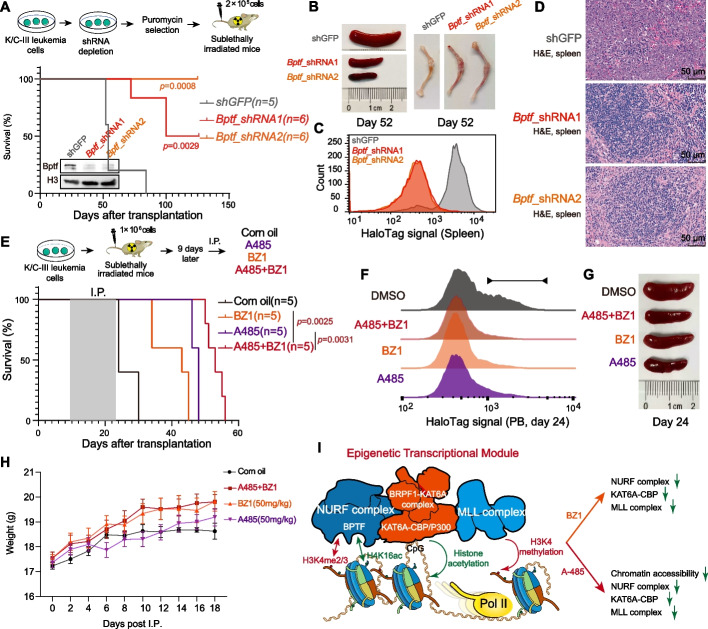
Therapeutic targeting of KAT6A-rearranged leukemia with BPTF and CBP/P300 inhibitors **A** Kaplan–Meier survival analysis of mice injected with K/C-III leukemic splenocytes with or without *Bptf* knockdown. *Bptf* depletion significantly extends survival (*n* = 5–6 mice per group; log-rank test). **B** Endpoint analysis shows reduced leukemia progression in recipient mice with *Bptf* knockdown. **C** Quantification of HaloTag^+^ leukemia cells in the spleen of recipient mice demonstrates a reduced leukemia burden in the *Bptf*-depleted group. **D** H&E-stained spleen Sects. (20 × magnification) reveal preserved spleen architecture in mice with *Bptf* knockdown. **E** In vivo treatment model for K/C-III leukemia using BZ1 and A485. Mice were treated with vehicle, BZ1 (50 mg/kg), A485 (50 mg/kg), or their combination via intraperitoneal injections for 10 times. Kaplan–Meier survival curves indicate prolonged survival in all treatment groups, with the combination therapy showing the most pronounced effect (log-rank test). **F**, **G** Post-treatment leukemia burden analysis: (F) Percentage of HaloTag^+^ leukemia cells in peripheral blood and (G) spleen measured on day 24 post-engraftment, showing reduced leukemia burden with BZ1, A485, and combination treatments. **H** Body weight monitoring of K/C-III leukemia mice during treatment. BZ1 (50 mg/kg), A485 (50 mg/kg), and combination treatment were well-tolerated, with no significant weight loss observed. **I** Proposed model for the self-reinforcing KAT6A-NURF-MLL module in KAT6A-rearranged leukemia. KAT6A associates preferentially with H3K4me2/3-marked chromatin and interacts with the NURF complex, a reader of H3K4me2/3. This interaction enhances KAT6A’s chromatin association, while the NURF complex further interacts with the MLL/COMPASS complex to sustain H3K4 methylation. CBP/P300-mediated histone acetylation enhances chromatin accessibility, enhancing the KAT6A-NURF-MLL module at target genomic loci

Building on the inhibitory effects of BZ1 in KAT6A-rearranged leukemia cells, we evaluated the therapeutic potential of targeting BPTF in the K/C leukemia model. BPTF was depleted in K/C-III leukemia cells using shRNA, and 2 × 10^5^ cells were transplanted into recipient mice for secondary transplantation (Fig. 7A). Disease monitoring revealed that BPTF knockdown significantly delayed leukemia progression compared to GFP-shRNA (shGFP) controls. By day 52 post-transplantation, mice receiving shGFP cells displayed advanced leukemia symptoms, including splenomegaly and pale bone marrow (Fig. 7B). In contrast, recipients of BPTF-depleted cells exhibited no such abnormalities. Quantitative analysis showed a marked enrichment of HaloTag-positive leukemia cells in the shGFP group, which was nearly absent in the BPTF knockdown group (Fig. 7C). Histological analysis confirmed disrupted splenic architecture in GFP-shRNA mice, while spleens from BPTF-depleted mice retained normal morphology (Fig. 7D). BPTF depletion also reduced colony formation in KAT6A-NCOA3A and KAT6A-TIF2 leukemic cells versus controls (Additional file 1: Fig. S9A and B), confirming that NURF dependency is conserved across KAT6A fusion subtypes. These results underscore the essential role of BPTF in K/C leukemia progression and its potential as a therapeutic target.

To further explore therapeutic strategies, we evaluated the efficacy of small-molecule inhibitors targeting BPTF (BZ1) and CBP/P300 (A485) in a K/C-III secondary transplantation model (Fig. 7E). Mice were injected with 8 × 10^5^ leukemia cells and treated intraperitoneally with vehicle (corn oil), BZ1 (50 mg/kg), A485 (50 mg/kg), or a combination of both inhibitors. Kaplan–Meier survival analysis demonstrated that both BZ1 and A485 prolonged survival (Fig. 7E), reduced peripheral blood leukemic blasts (Fig. 7F), and decreased spleen volumes by day 24 after tail veil injection (Fig. 7G). Notably, the combination treatment exhibited superior efficacy in delaying leukemia progression compared to either treatment alone (Fig. 7E). Importantly, all treatments were well-tolerated, with no significant body weight loss observed (Fig. 7H).

To determine whether their combined effects are synergistic or additive, we conducted co-treatment with BZ1 (0.5–2 μM) and A-485 (0.5–1 μM) in K/C-III leukemic cells, which yielded a Bliss synergy score of − 0.11 (Additional file 1: Fig. S9C), indicating additive effects (score ≈ 0) rather than synergy (score > 10). Besides, BZ1 co-treatment did not further reduce the population of c-Kit^+^CD34^+^ leukemic cells compared to A485 treatment alone (Additional file 1: Fig. S9D), suggesting no enhanced differentiation effect. Moreover, RNA-seq of K/C-III leukemic cells treated with low-dose BZ1 (1 μM) and A485 (0.5 μM) revealed that co-treated cells shared most of the downregulated genes with A485-treated cells and no unique pathways or gene sets were specifically suppressed in co-treated cells (Additional file 1: Fig. S9E). These data demonstrate that BZ1 and A485 exert additive, not synergistic, antileukemic effects in K/C-III leukemic cells. The absence of unique transcriptional programs or enhanced differentiation in co-treated cells may reflect their shared mechanistic targeting of the KAT6A-NURF-MLL module. Furthermore, we evaluated the therapeutic potential of BZ1 and A485 in common non-KAT6A-rearranged human leukemia cell lines and found that that BZ1 and A485 do not broadly inhibit leukemia cell proliferation but exhibit selective efficacy (Additional file 1: Fig. S10A and B). Together, these findings underscore the critical oncogenic dependency of KAT6A-rearranged leukemia on BPTF and CBP/P300. They validate these factors as promising therapeutic targets, with the combination approach offering enhanced efficacy for treating this rare but aggressive leukemia subtype.

## Discussion

This study addresses the longstanding challenge of studying large oncogenic fusion proteins by employing a domain-focused truncation strategy. This innovative approach enabled the generation of robust murine AML models for K/C and K/P fusions, faithfully recapitulating the transcriptomic, morphological, and immunophenotypic characteristics of KAT6A-rearranged AML. Beyond their immediate relevance to KAT6A fusions, these models offer a scalable framework for investigating other large oncogenic fusions, broadening therapeutic insights across diverse AML subtypes. We demonstrate that KAT6A preferentially associates with H3K4me2/3-marked chromatin and interacts with the NURF complex, a known reader of these marks [35, 42]. This interaction enhances KAT6A’s chromatin association and works with the MLL/COMPASS complex to sustain H3K4 methylation. Additionally, CBP/P300 catalyzes histone acetylation at multiple sites, including H4K16ac and H3K27ac, thereby enhancing chromatin accessibility and facilitating the recruitment of the KAT6A-NURF-MLL module to targeted genomic loci. Collectively, these findings define a critical self-reinforcing module that underpins the leukemogenic activity of K/C and K/P fusions (Fig. 7I).

KAT6A, a critical acetyltransferase, operates within protein complexes including BRPF1, ING4/5, and MEAF6, playing pivotal roles in development and hematopoiesis [10, 13, 43]. Interestingly, while KAT6A’s catalytic activity supports acetylation of marks like H3K9ac, H3K23ac, and H3K23 propionylation [44, 45], deletion of the MYST domain, but not its catalytic activity, abolishes colony formation and leukemogenesis in mouse AML models (21). Consistent with this, our results demonstrate that catalytically inactive KAT6A retains genomic occupancy comparable to its wild-type form. This underscores that KAT6A’s chromatin-binding ability is independent of its enzymatic activity. Furthermore, we identify the NURF complex as a key interactor of the KAT6A MYST domain, facilitating its genomic occupancy. This interaction underscores the critical role of structural partnerships over KAT6A enzymatic activity in leukemogenesis, unveiling a KAT6A catalytic-independent mechanism through which KAT6A fusions contributes to AML pathogenesis.

Beyond serving as an H3K4me2/3 reader, the NURF complex facilitates transcriptional regulation by catalyzing nucleosome sliding at promoters, thereby enhancing transcriptional access [37, 46, 47]. Disrupting this process using the small-molecule inhibitor BZ1 reduces BPTF-chromatin interactions by ~ 50%, yet ChIP-MNase and ATAC-seq analyses reveal no significant changes in nucleosome positioning and chromatin accessibility at promoters. These findings suggest that partial NURF disruption is insufficient to induce detectable nucleosome repositioning, highlighting the resilience of chromatin architecture. These observations illuminate the nuanced role of NURF in maintaining the oncogenic epigenetic landscape in AML and emphasize the need for further exploration into the thresholds and dynamics of NURF activity. The partial displacement of the fusion by BPTF inhibition may disrupt proliferative programs but retains residual fusion activity sufficient to maintain the differentiation blockade. In contrast, A485 could directly inhibits the fusion’s enzymatic activity, eliminating both proliferation- and differentiation-blocking signals, thereby enabling cell differentiation.

In contrast to KAT6A’s acetylation activity, CBP/P300 HAT activity is indeed indispensable for KAT6A-rearranged AML. CBP/P300 catalyzes histone acetylation at multiple sites, contributing to a dynamic and extensive acetylome [19, 48]. Acetylation marks likely enhance chromatin accessibility, facilitating RNA Pol II loading and the recruitment of the KAT6A-NURF-MLL module to chromatin. Besides, the bromodomain of BPTF, a core component of the NURF complex, specifically recognizes H4K16ac, while its PHD domain binds H3K4me2/3 [35, 36, 49]. These interactions establish a unique trans-histone modification pattern within a single nucleosome, where BPTF’s bivalent binding co-localizes with these marks across the genome [49]. Such co-localization suggests a coordinated mechanism whereby distinct histone modifications converge to regulate chromatin dynamics and selectively recruit the KAT6A-NURF-MLL module. This interplay likely enhances the genomic specificity of KAT6A fusions through the combined actions of histone reader proteins and modifying enzymes. These findings provide deeper insights into how chromatin architecture and histone modifications orchestrate oncogenic transcriptional programs, while also uncovering critical dependencies that may serve as therapeutic vulnerabilities in KAT6A-rearranged AML.

Our findings address a critical challenge in treating KAT6A-rearranged AML, a high-risk subtype with poor prognosis [7], by highlighting the therapeutic potential of targeting BPTF and CBP/P300 activity. BZ1, a BPTF-H4K16ac inhibitor, induces cell cycle arrest and suppresses leukemia cell proliferation but exhibits weaker efficacy than BPTF knockdown, highlighting the need for further optimization. In contrast, the acetyltransferase inhibitor A485 effectively promotes leukemia cell differentiation. The differential effects of BZ1 and A485 may stem from A485’s inhibition of CBP/P300 acetyltransferase activity, leading to a rapid and dramatic reduction in chromatin accessibility, or its stronger suppression of K/C-III, BPTF, and MLL occupancy. Notably, the combination of A485 and BZ1 yields superior therapeutic outcomes, emphasizing the potential of dual targeting within the KAT6A-NURF-MLL module for KAT6A-rearranged AML. Future studies should evaluate the therapeutic efficacy of targeting this module—both as single agents and in combination therapies—in primary AML patient-derived samples to advance their clinical potential. Moreover, we acknowledge that our animal models are based on overexpression of truncates of KAT6A chimeras, which may lead KAT6A chimeras to bind sites that might not be occupied in a more physiological setting or potentially exclude critical genomic binding sites or protein interactions. Collectively, by elucidating the interplay between chromatin dynamics, histone modifications, and transcriptional regulation in KAT6A-rearranged AML, this study provides a foundation for innovative therapeutic strategies targeting epigenetic vulnerabilities in this rare but aggressive leukemia subtype.

## Conclusions

In summary, we established murine leukemia models for large KAT6A fusions and characterized their morphological, immunophenotypic, and transcriptomic features. We found that KAT6A chimeras colocalize with H3K4me2/3-marked regions and interact with the H3K4me2/3 reader complex NURF. Furthermore, we identified a self-reinforcing epigenetic module involving KAT6A chimeras, NURF, and MLL/COMPASS, which sustains leukemogenic transcriptional programs through H3K4me2 deposition and histone acetylation. Targeting this module via NURF or CBP/P300 inhibition demonstrates efficacy in K/C leukemia models, with enhanced therapeutic effects when combined. More importantly, our findings establish robust models for investigating disease mechanisms and therapeutic strategies, offering broader implications for epigenetics and blood cancers.

## Methods

### Cell culture and DNA construction

HEK293T (ATCC CRL-3216) and Mouse Embryonic Fibroblast (MEF; ATCC CRL-2752) cells were cultured in high-glucose DMEM (Life Technologies) supplemented with 10% FBS (LONSERA S711-001), 1% penicillin/streptomycin (Gibco), and L-glutamine (Gibco) at 37 °C in a 5% CO_2_ atmosphere. Drosophila S2 cells (Gibco R69007) from Invitrogen were maintained in Schneider's medium (Sigma-Aldrich) at 25 °C. Cell lines were routinely tested for mycoplasma contamination using MycoAlert (Lonza) and authenticated by DNA fingerprinting (ATCC). Primary murine HSPCs, isolated using CD117 MicroBeads, and K/C-III and K/P-III leukemia cells were cultured in Retronectin-coated plates (Takata T100A) with RPMI 1640 medium (Gibco) supplemented with 1% penicillin/streptomycin, 10% FBS (Gibco), and murine SCF, IL-6, and IL-3. Cell viability was assessed with a Bio-Rad TC20 automated cell counter.

KAT6A truncates, K/C, and K/P fusion constructs were cloned into the MSCV retroviral vector (Addgene #68,469), incorporating N-terminal Flag and HaloTag tags for detection and purification purposes. Detailed sequences of these constructs are provided in Additional file 2: Table S6. For shRNA targeting human or mouse *Bptf*, oligonucleotides were designed and cloned into the PLKO shRNA vector. The sequences for the shRNA constructs are listed in Additional file 2: Table S7.

### Generation of Antibodies

Antibodies were generated by immunizing New Zealand White rabbits with recombinant proteins. Anti-BPTF serum was produced by immunizing rabbits with a BPTF fragment (residues 121–340) purified from *E. coli* BL21 cells. Similarly, anti-HaloTag serum was raised using recombinant HaloTag protein purified from *E. coli* BL21 cells. The serum was subsequently aliquoted and stored at −20 °C or lower for long-term use. Commercial antibodies were listed in Additional file 2: Table S8.

### Isolation of c-Kit-enriched HSPCs

Bone marrow was collected from the femurs and tibias of 6-week-old female C57BL/6 J mice. Cells were flushed from the bones with ice-cold PBS using a 25-gauge needle and syringe. The suspension was filtered through a 40-µm nylon mesh cell strainer to remove debris and aggregates. Red blood cells were lysed with Erythrocyte Lysate for 2 min at 4 °C. HSPCs were enriched using CD117 MicroBeads Kit (Miltenyi Biotec) and magnetic-activated cell sorting. Cells were incubated with CD117 beads for 15 min at 4 °C with gentle rotation, followed by magnetic separation using a Miltenyi Biotec column per the manufacturer’s instructions. Isolated HSPCs were either used for transcriptome analysis or cultured.

### Retrovirus preparation and HSPCs transduction

HEK293T cells were transfected with an MSCV retroviral vector containing various HaloTag-KAT6A fusions, along with the pCL-Eco retrovirus packaging vector (Addgene #12,371), using Polyethyleneimine. After 48 h, the retroviral supernatant was collected, filtered through a 0.45-µm filter, and concentrated by centrifugation at 18,000 rpm for 90 min at 4 °C in a Himac CR21N rotor using Beckman Ultra-Clear tubes. The viral pellet was used immediately for transduction. c-Kit-enriched HSPCs were cultured on RetroNectin-coated plates (Takata T100A) and underwent spinoculation at 1,000 g at 37 °C for 60 min, then incubated for 24 h. After spinoculation, cells were resuspended in fresh cytokine-supplemented medium. Transduction efficiency was assessed by flow cytometry (Beckman Coulter CytoFLEX S) to detect HaloTag-tagged protein expression.

### Murine K/C and K/P leukemia models

Transduced HSPCs (5 × 10^6^ cells) were intravenously injected into the tail vein of primary recipient female C57BL/6 mice (8 weeks old) that had been previously irradiated with 900 cGy. The mice were monitored closely for signs of acute leukemia, including weight loss, reduced mobility, malaise, splenomegaly, and a hunched posture. Upon observing clear symptoms of leukemia, the mice were euthanized, and their spleens and bone marrow were harvested for subsequent analyses. Spleen homogenates were cultured in RPMI 1640 supplemented with 1% penicillin/streptomycin, 10% FBS, murine SCF, IL-6, and IL-3. For secondary transplantation, 2 × 10^5^ leukemia cells from primary recipients were injected into sublethally irradiated (450 cGy) female C57BL/6 mice (8–10 weeks old). Kaplan–Meier survival analysis was conducted using the log-rank test in GraphPad Prism.

### Giemsa-Wright staining and H&E staining

Wright-Giemsa staining was performed on murine AML cells harvested from the bone marrow of recipient mice. Blast cells and differentiated leukemia cells were visualized and photographed using a ZEISS Axio Imager 2 microscope with a 40 × objective lens. For histological examination, mouse spleens were dissected, fixed in 4% paraformaldehyde (PFA), and embedded in paraffin. Paraffin-embedded spleen sections were stained with hematoxylin and eosin (H&E) and analyzed for AML cell infiltration.

### RT-qPCR

Murine MP cells (Lin^−^c-Kit^+^) were sorted by flow cytometry. RNA was extracted from murine leukemia cells, MP cells, or bone marrow cells and reverse transcribed using ReverTra Ace™ qPCR RT Master Mix (Toyobo). RT‒qPCR was conducted using SYBR Green Master Mix (Vazyme). The sequences of primers used are provided in Additional file 2: Table S9.

### In Vivo Treatment with BPTF and CBP/P300 Inhibitors

To evaluate the therapeutic effects of BPTF and CBP/P300 inhibitors, 8 × 10^5^ K/C-III leukemia cells were transplanted into sublethally irradiated (450 cGy) 8-week-old female C57BL/6 mice. BPTF inhibitor BZ1 (MCE HY-132889) and CBP/P300 inhibitor A485 (Aladdin A287597) were dissolved in corn oil. Nine days post-transplantation, mice received intraperitoneal injections of either vehicle, 50 mg/kg BZ1, 50 mg/kg A485, or both inhibitors for 10 times. Body weights and overall survival rates of the recipient mice were monitored and compared across treatment groups.

### Spectral Flow Cytometry

Spectral flow cytometry was conducted using an adapted protocol [33]. The details of antibodies are provided in Additional file 2: Table S8. Data acquisition was performed on a 4-laser Cytek Aurora spectral flow cytometer, capturing approximately 1 × 10^7^ events. SpectroFlo software (Cytek Biosciences) was used for analysis, employing ordinary least squares linear unmixing. Data visualization was carried out using the OMIQ cytometry analysis platform. For lineage-specific marker analysis, leukemia cells were incubated with individual antibodies targeting lineage markers (Ter-119, Mac1, Gr-1, B220, CD5, CD3, CD4, and CD8), stained with F(ab')2-IgG, detected via flow cytometry, and analyzed with FlowJo software to generate histograms.

### Analysis for clinical data

For the analysis of KAT6A-rearranged AML patients, RNA expression data were extracted from the TCGA-LAML database (https://portal.gdc.cancer.gov). AML patients were classified into two subgroups based on KAT6A rearrangement or normal karyotypes based on clinical profiles from the University of California, Santa Cruz (UCSC) Xena project (TCGA) Acute Myeloid Leukemia (LAML), http://xena.ucsc.edu). To investigate the gene expression signatures associated with KAT6A rearrangement, upregulated signature genes were obtained from a previously published study [8]. GSEA was then applied to test the expression of this signature in the two patient groups, allowing for comparison between KAT6A-rearranged and normal karyotype AML patients.

### CFU, proliferation, and cell cycle analysis

For the CFU assay, 1 × 10^4^ leukemic cells were cultured in M3434 medium in 12-well plates. After 7 days of incubation, colonies containing more than 10 cells were counted using an inverted tissue culture microscope. In the proliferation assay, 1 × 10^5^ leukemic cells from murine models were seeded into 96-well plates and treated with DMSO or the BPTF inhibitor for 4 days. Cell viability was assessed using the Promega CellTiter-Glo® Luminescent Cell Viability Assay according to the manufacturer's protocol. Bliss synergy score was calculated by SynergyFinder web version 3.0 (https://synergyfinder.fimm.fi). For cell cycle analysis, leukemia cells were harvested, washed once with pre-cooled PBS, and fixed overnight in pre-cooled 70% ethanol. The fixed cells were stained with a propidium iodide solution containing 10% Triton X-100, 10 mg/mL RNase A, and 1 mg/mL propidium iodide. Following a 15-min incubation at 37 °C, cell cycle distribution was analyzed using a CytoFLEX S flow cytometer.

### Cell differentiation assays

5 × 10^6^ splenocytes from leukemia mice were treated with DMSO or A485 (1 μM) for one week. For Giemsa-Wright staining, 1 × 10^6^ cells were seeded on coverslips and photographed using a ZEISS Axio Imager 2 microscope. For flow cytometry analysis, cells were washed with PBS and stained with Biolegend antibodies (Lin, CD127, Sca1, CD34, c-Kit, FcγR), and analyzed by spectral flow cytometry. The L-GMP cell marker profile was used as CD127^−^Sca1^−^CD34^+^c-Kit^+^FcγR^+^Lin^−^.

### IP-MS

HEK293T cells transfected with either KAT6A N-terminus or KAT6A fusion constructs were grown in five 150 mm dishes. After harvesting, the cells were resuspended in Dignam buffer (10 mM Tris–HCl pH 7.9, 1.5 mM MgCl_2_, 10 mM KCl, with protease inhibitors) and washed once in the same buffer. The cell pellets were centrifuged at 600 g for 5 min at 4 °C, and the supernatant was discarded. Cells were then resuspended in MRIPA buffer (25 mM Tris–HCl, pH 7.4, 1% NP-40, 0.25% Na-deoxycholate, 300 mM NaCl, 5% glycerol) with Benzonase and MgCl_2_ to lyse the nuclei at 4 °C for 30 min. Following nuclear lysis, the lysates were centrifuged at 13,500 rpm for 30 min at 4 °C. The supernatants were incubated overnight at 4 °C with anti-FLAG magnetic beads for protein capture. The bead-protein complexes were then washed three times with MRIPA buffer. Purified proteins were eluted with 0.1 M glycine (pH 2.0), with 10% of the eluted samples analyzed by silver staining.

The remaining protein samples were precipitated by adding pre-cooled acetone at −20 °C overnight. The samples were then alkylated with iodoacetamide and digested with trypsin (Promega V528A) for 16 h at 37 °C. Digestion was stopped with 10% TFA, and peptides were desalted using C18 StageTips. Mass spectrometry analysis was conducted using an Orbitrap Exploris 480 equipped with a FAIMS Pro interface. Data processing was performed with Proteome Discoverer 2.4, with a four-stage program. Proteins with a q-value ≤ 0.01 were assigned high confidence, while those with a q-value ≤ 0.05 were assigned medium confidence.

### BPTF depletion and chromatin extraction

HEK293T cells were infected with lentivirus encoding shRNA sequences targeting human BPTF (GTC TAC CAC AAT CAA TAC TC or CAG GCA ATA CTA ATG TGA AC) and subsequently selected with puromycin for two days. Cells were then harvested, and cell membrane lysis was carried out in 0.5 mL of ice-cold E1 buffer (50 mM HEPES–KOH, pH 7.5, 140 mM NaCl, 1 mM EDTA, 10% glycerol, 0.5% NP-40, 0.25% Triton X-100, 1 mM DTT, and protease inhibitors). The nuclear pellet was isolated by centrifugation and resuspended in 0.5 mL of ice-cold E2 buffer (10 mM Tris–HCl, 200 mM NaCl, 1 mM EDTA, 0.5 mM EGTA, and protease inhibitors) to further purify the nuclear fraction. After discarding the supernatant, the pellet was resuspended in 100 μL of ice-cold E3 buffer (50 mM Tris–HCl, 20 mM NaCl, 1 mM MgCl_2_, 1% NP-40, and protease inhibitors). Benzonase was added at a 1:1,000 dilution to digest nucleic acids, and the solution was rotated for 30 min at 4 °C to ensure thorough digestion. Following a final centrifugation at 16,000 g for 10 min at 4 °C, the supernatant containing the chromatin fractions was collected for further analyses.

### Western blot analysis

Cells or chromatin fractions were lysed in 1 × SDS loading buffer and boiled at 100 °C for 5 min. Equal amounts of protein from whole-cell lysates or chromatin fractions were then resolved by SDS-PAGE and transferred onto nitrocellulose membranes for immunoblotting. Band intensity was quantified using ImageJ (NIH) and normalized to histone H3 as loading controls. Uncropped Western blot and gel images were provided in Additional file 3.

### CUT&RUN analysis

CUT&RUN reactions were performed as described [50], following the “Standard CUT&RUN” protocol. Briefly, freshly harvested cells (1 × 10^6^) were bound to CoA beads 4FF (Smart Lifesciences), resuspended in antibody binding buffer, and incubated overnight with primary antibodies. CUT&RUN experiments with leukemia cells were performed using ex vivo-isolated AML cells from leukemic mice. The samples were then washed and bound to homemade pA/G-MNase. Chromatin digestion was initiated by the addition of CaCl_2_ and stopped after 30 min with STOP buffer containing chelating agents. Samples were incubated for 30 min at 37 °C to release CUT&RUN fragments and then treated for 1 h at 50 °C with proteinase K. A purification step was performed using SRPI beads. Library preparation was done using the NEBNext Ultra II DNA Library Prep Kit for Illumina, followed by sequencing on a NovaSeq 6000 to achieve a depth of at least 10 million paired-end 150 bp reads per sample. CUT&RUN reads were aligned to the human or mouse genome with Bowtie2 with the following options: —very-sensitive —no-discordant —no-mixed –X 700 –dovetail. Peak calling was performed with MACS2 using IgG samples as background. Coverage signal profiles in bigwig format were generated using the bamCoverage function from deepTools with a Counts-Per-Million per-sample normalization. These profiles were visualized using deepTools computeMatrix and plotHeatmap functions.

### ChIP-seq

ChIP-seq was performed as previously described [51, 52]. Library preparation was carried out using the NEBNext Ultra II DNA Library Prep Kit for Illumina, followed by sequencing on a NovaSeq 6000. ChIP-seq reads were aligned to the human or mouse genome using Bowtie version 1.1.2. Only uniquely mapping reads with up to two mismatches within the 150 bp reads were considered for further analysis.

### ChIP-MNase

ChIP-MNase was performed following an established protocol [53]. Briefly, HEK293T cells expressing K/C-III were fixed with 4% formaldehyde for 10 min at room temperature and then quenched with glycine. The fixed cells were lysed in L1 buffer (50 mM Tris–HCl pH 8.0, 2 mM EDTA, 0.1% NP-40, 10% glycerol, 1 × protease inhibitors, 1 mM PMSF, 2 mM DTT) on ice for 5 min. Following centrifugation, the nuclear pellet was resuspended in ice-cold wash buffer D (50 mM Tris–HCl pH 8.0, 0.1 mM EDTA, 5 mM Mg acetate, 5 mM DTT, 25% glycerol) and incubated for 5 min before centrifuging at 2,000 g for 5 min. The pellet was resuspended in 400 μL MNase buffer (10 mM Tris–HCl pH 7.4, 15 mM NaCl, 60 mM KCl, 1 mM CaCl_2_, 250 mM sucrose, 0.5 mM DTT) and underwent two freeze–thaw cycles in liquid nitrogen to disrupt nuclei. Chromatin was sonicated with a Diagenode Bruptor Plus (low-power mode, 8 cycles of 30 s ON and 30 s OFF).

Next, 2 U of MNase was used to digest fixed chromatin at 25 °C for 30 min with shaking at 1200 rpm. Digestion was halted with EDTA, and 1 mL of buffer V (50 mM Tris pH 7.4, 50 mM NaCl, 5 mM EDTA) was added. The supernatant was pre-cleaned with rProtein A/G beads (Smart Lifesciences, SA032005). Samples were incubated overnight at 4 °C with 5 μg H3K4me2 antibody and 15 μL rProtein A/G beads. Beads were washed three times with MNase buffer, followed by a second MNase digestion (15 μL MNase buffer containing 2 U MNase) at 25 °C for 30 min with shaking. The reaction was stopped with 34 mM EDTA and treated with proteinase K. DNA was purified by phenol–chloroform extraction and ethanol precipitation, and ChIP-MNase libraries were prepared using the NEBNext Ultra II DNA library prep kit for Illumina.

### ATAC-seq

Standard libraries were prepared following a previously established protocol [54, 55]. Briefly, 2.5 × 10^4^ mouse leukemia cells were treated and centrifuged at 600 g for 5 min at room temperature for each reaction. The cell pellet was resuspended in 50 μL lysis buffer containing 10 mM Tris–HCl (pH 7.5), 10 mM NaCl, 3 mM MgCl_2_, 0.1% NP-40, 0.1% Tween-20, and 0.02% digitonin, and centrifuged at 500 g for 10 min at 4 °C. After centrifugation, the cell pellet was processed immediately for the transposition reaction, resuspended in 50 μL transposase mixture containing 25 μL 2 × TD buffer, 22.5 μL nuclease-free water, and 2.5 μL Tn5 transposase. The mixture was incubated for 30 min at 37 °C with mixing at 1,000 rpm. The transposed DNA was purified and amplified using NEBNext high-fidelity 2 × PCR master mix with custom Nextera PCR primers. The optimal number of PCR cycles was determined by qPCR, and the DNA libraries were size-selected and sequenced on the NovaSeq 6000 platform. ATAC-seq reads were aligned to the mouse genome, and alignments were processed with Bowtie using the command options -m 1 -v 2, allowing only uniquely mapping reads with up to two mismatches within the 150 bp reads. BigWig files were generated using the ‘bamCoverage’ function in Deeptools.

### Histone modification analysis

Histone modification analysis was conducted following a previously published protocol [56]. Briefly, 293 T cells were transfected with MSCV-KAT6A-CBP for 48 h, followed by treatment with DMSO or A485 for 0, 6, or 12 h. Cell pellets (~ 50 µL) were collected by centrifugation at 300 × g for 5 min and washed twice with PBS. The cell pellets were resuspended in Nuclear Isolation Buffer (NIB; 15 mM Tris, 60 mM KCl, 15 mM NaCl, 5 mM MgCl_2_, 1 mM CaCl_2_, 250 mM sucrose, pH 7.5) at a 1:10 (v/v) ratio and washed twice. The pellets were then resuspended in NIB containing 0.2% NP40, incubated on ice for 10 min, and centrifuged at 1,000 × g for 10 min at 4 °C. The nuclei were washed twice with NIB (without NP40) and resuspended in chilled 0.2 M H_2_SO4 at a 1:5 (v/v) ratio. Samples were incubated with gentle shaking at 4 °C for 4 h, followed by centrifugation at 3,400 × g for 5 min at 4 °C. The supernatant was collected and mixed with 100% TCA at a 1:3 (v/v) ratio, incubated on ice for 1 h, and centrifuged at 3,400 × g for 5 min.

The resulting pellet was washed once with ice-cold acetone containing 0.1% HCl and once with 100% ice-cold acetone. The pellet was dried using a vacuum centrifuge and dissolved in minimal ddH_2_O to completely solubilize the white layer. After centrifugation, the supernatant was collected, and protein concentrations were measured using a BCA assay. Histone proteins were analyzed by SDS-PAGE on 15% acrylamide gels. For chemical derivatization, histones were adjusted to 50 mM NH_4_HCO_3_ (pH 8.0), and a fresh propionylation reagent (propionic anhydride:acetonitrile, 1:3 v/v) was added at a 1:4 (v/v) ratio. NH_4_OH was used to re-establish pH 8.0, and the mixture was incubated at room temperature for 15 min. Samples were dried to 10–20 µL in a vacuum concentrator and diluted to a final volume of 40 µL with ddH_2_O. The propionylation step was repeated once. Histones were resuspended in 50 mM NH_4_HCO_3_ at a concentration of ≥ 1 µg/µL (pH 8.0) and digested with trypsin at a 1:20 (w/w) ratio overnight at 37 °C. Digestion was stopped by freezing at −80 °C, and samples were dried to 10–20 µL. Samples were resuspended in 30 µL of 100 mM NH_4_HCO3 and subjected to a second round of propionylation. Finally, samples were diluted with 50–100 µL of 0.1% TFA in ddH_2_O, desalted using C18 desalting tips, dried in a vacuum concentrator, and analyzed by LC-FAIMS-MS/MS.

### RNA-seq analysis

Mouse leukemia cells isolated from leukemic mice, MP cells (Lin-, c-Kit +), and c-Kit-enriched HSPCs were lysed with Trizol (Ambion 15,596,018) for total RNA extraction. mRNA from 2 µg of total RNA was enriched with VAHTS mRNA capture beads and fragmented to 200–500 nt lengths using 2 × ProtoScript II buffer. Strand-specific RNA-seq libraries were prepared and sequenced with 150 bp paired-end reads on the NovaSeq 6000 platform. The sequencing data were aligned to the mm10 mouse genome, normalized (RPM), and visualized using the UCSC genome browser. Differential gene expression analysis was performed with HTSeq-count (version 0.12.3) and DESeq2 (version 1.32.0) [57]. Gene ontology enrichment analysis was conducted using Metascape.

### Statistical analysis

Statistical analysis was performed using GraphPad Prism 8 software. Data are expressed as Mean ± SD. The statistical tests employed, including one-way ANOVA, Student's t-test, and log-rank test, were chosen based on the experimental design and data characteristics. Statistical significance was set at *P* < 0.05 (*, *P* < 0.05; **, *P* < 0.01; ***, *P* < 0.001).

### Data and materials availability

All data are available in the main text or the supplementary materials. The H3K9ac and H3K27me3 ChIP-seq data from 293 T cells were originally deposited into GEO under the accession number: SRR19676715 [25] and SRR9604322 [58]. The H3K9me3 293 T CUT&RUN were obtained under the accession number: SRR21837874 and SRR21862863 [59]. The raw and processed mRNA-seq, ATAC-seq, and CUT&RUN datasets supporting the conclusions of this article have been deposited in the Gene Expression Omnibus (GEO) repository under accession numbers: GSE299379 [60], GSE299380 [61], GSE299381 [62], GSE299382 [63], and GSE299383 [64]. The mass spectrometry proteomics data generated in this study have been submitted to the ProteomeXchange Consortium via the PRIDE partner repository under the dataset identifier PXD061491 [65] and PXD061565 [66]. All unique materials generated are available from the corresponding author upon reasonable request. No custom scripts and software were used besides those mentioned in the Methods section.

## Supplementary Information

Additional file 1: Figures S1-S10

Additional file 2: Tables S1-S9. Table S1. mRNA-seq analysis of c-Kit^+^ bone marrow, MPs, K/C-III, and K/P-III leukemia cells. Table S2. IP-MS analysis of KAT6A and KAT6A fusions. Table S3. mRNA-seq analysis of K/C-III leukemia cells after BZ1 treatments. Table S4. mRNA-seq analysis of K/C-III leukemia cells after A485 treatments. Table S5. List of 770 core target genes of K/C-III. Table S6. List of KAT6A truncates and KAT6A fusion sequence. Table S7. Primer sequences of BPTF shRNA. Table S8. List of antibodies used in this study. Table S9. List of RT-qPCR primers.

Additional file 3: Uncropped western blot and gel images

## Data Availability

All data are available in the main text or the supplementary materials. The H3K9ac and H3K27me3 ChIP-seq data from 293 T cells were originally deposited into GEO under the accession number: SRR19676715 [25] and SRR9604322 [58]. The H3K9me3 293 T CUT&RUN were obtained under the accession number: SRR21837874 and SRR21862863 [59]. The raw and processed mRNA-seq, ATAC-seq, and CUT&RUN datasets supporting the conclusions of this article have been deposited in the Gene Expression Omnibus (GEO) repository under accession numbers: GSE299379 [60], GSE299380 [61], GSE299381 [62], GSE299382 [63], and GSE299383 [64]. The mass spectrometry proteomics data generated in this study have been submitted to the ProteomeXchange Consortium via the PRIDE partner repository under the dataset identifier PXD061491 [65] and PXD061565 [66]. All unique materials generated are available from the corresponding author upon reasonable request. No custom scripts and software were used besides those mentioned in the Methods section.
