# Supplementary material for: KAT6A chimeras form a self-reinforcing epigenetic module with NURF and MLL/COMPASS to sustain AML
Source: Genome Biol. 2025 Aug 19;26:253. doi: 10.1186/s13059-025-03743-y (PMC12366150; doi:10.1186/s13059-025-03743-y)
Supplement: Supplementary file 1 — Additional file 1: Figures S1-S10 [file 13059_2025_3743_MOESM1_ESM.docx]

Additional file 1: Fig. S1-S10 and figure legends

Fig. S1.


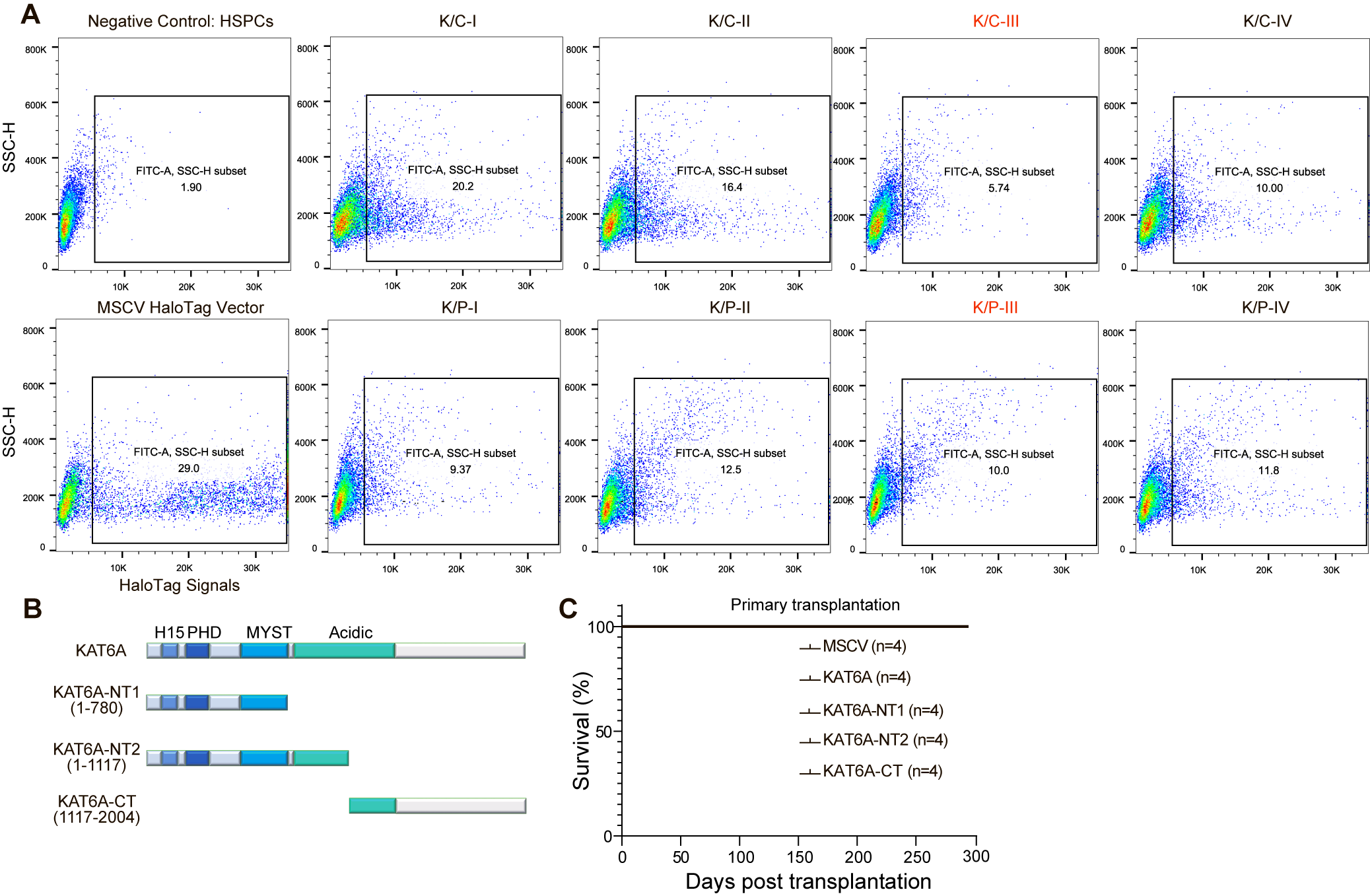


**Fig. S1. Retrovirus Infection of HSPCs by K/C and K/P truncation constructs.**

**A.** c-Kit-sorted murine bone marrow cells were molecularly tracked by incorporating HaloTag and transduced in RetroNectin-coated dishes via spinoculation to enhance transduction efficiency. Transduction efficacy was assessed using HaloTag R110 staining and flow cytometry analysis, revealing 5-20% transduction efficiency.

**B, C.** Design of KAT6A and KAT6A truncations. MSCV, KAT6A, and its truncations exhibited no transformation capability in c-Kit-sorted bone marrow cells.

Fig. S2.

**Fig. S2. Spectral flow cytometry analysis of K/C-III and K/P-III leukemia cells.**

**A, B.** Spectral flow cytometry analysis of K/C-III and K/P-III leukemia cells. The leukemia cell populations are indicated by arrows, highlighting their distinct cell surface marker profiles compared to normal murine hematopoietic cells. Notably, K/C-III and K/P-III leukemia cells exhibit similar surface marker expressions.

Fig. S3.

**
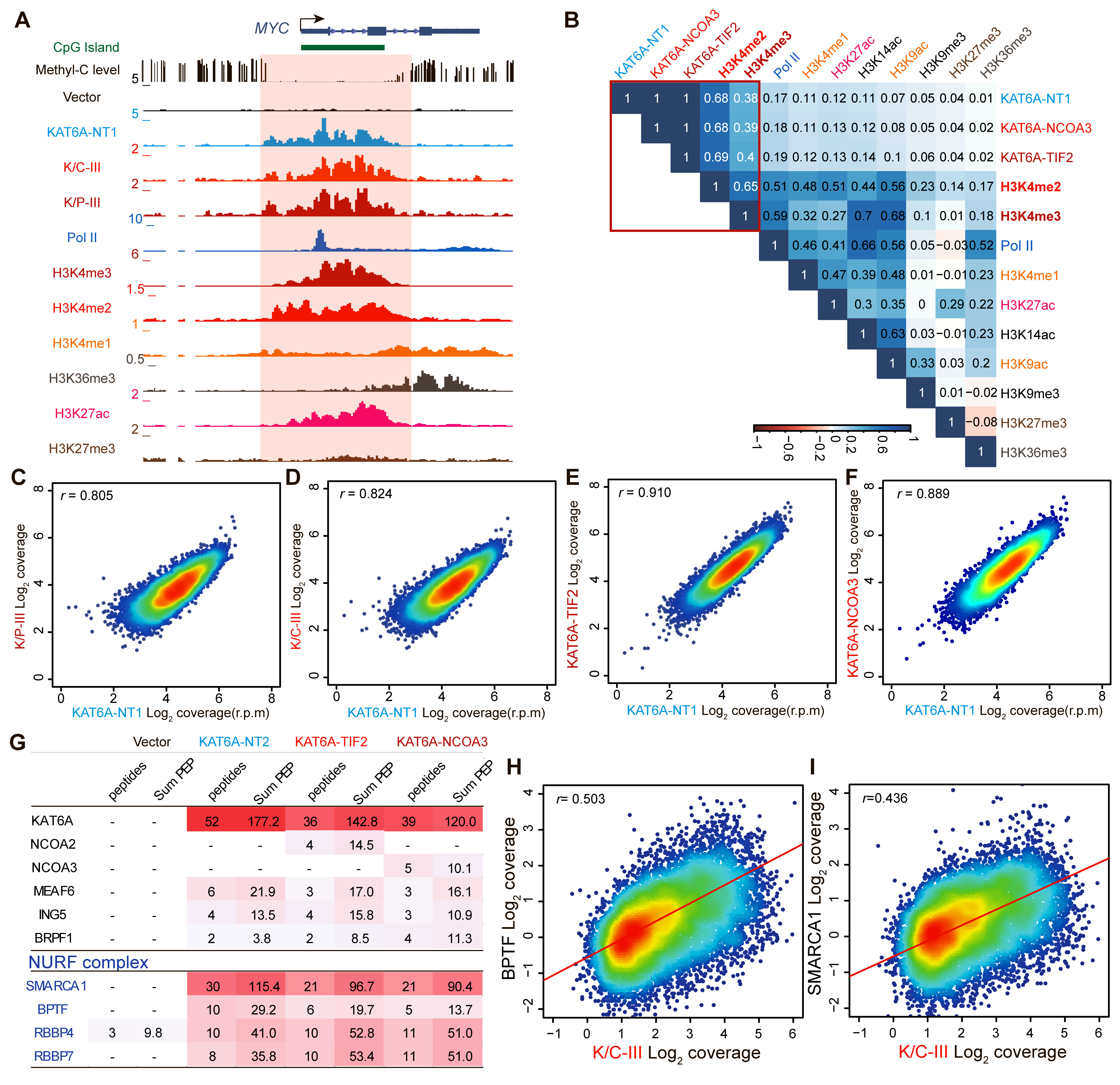
**

**Fig. S3. Interaction between the NURF complex and KAT6A.**

**A.** Genome browser tracks illustrating the distribution of CpG islands, methyl-C levels, KAT6A-NT1, K/C-III, K/P-III, Pol II, and histone markers in HEK293T cells. KAT6A-NT1, K/C-III, and K/P-III exhibit occupancy that extends beyond CpG islands, overlapping with H3K4me3 and H3K4me2 at the *MYC* promoter region.

**B.** Pearson correlation analysis demonstrating positive correlations between KAT6A, KAT6A-NCOA3, and KAT6A-TIF2 with H3K4me2 and H3K4me3 across the genome. KAT6A-NT1 peaks (N=10,770) were utilized for this genome-wide analysis.

**C-F,** Dot plot analyses comparing KAT6A-NT1 occupancy with CUT&RUN signals from K/P-III, K/C-III, KAT6A-NCOA3, and KAT6A-TIF2 in HEK293T cells.

**G,** Mass spectrometry analysis of KAT6A-NT2, KAT6A-NCOA3, and KAT6A-TIF2 immunoprecipitates, showing the peptide and sum PEP score for the BRPF1-KAT6A complex and NURF complex subunits. The MSCV vector serves as the binding control.

**H, I.** Positive correlation of BPTF and SMARCA1 CUT&RUN signals with K/C-III occupancy in K/C-III leukemia cells. Pearson correlation analysis was performed, with the *r* values presented.

Fig. S4.


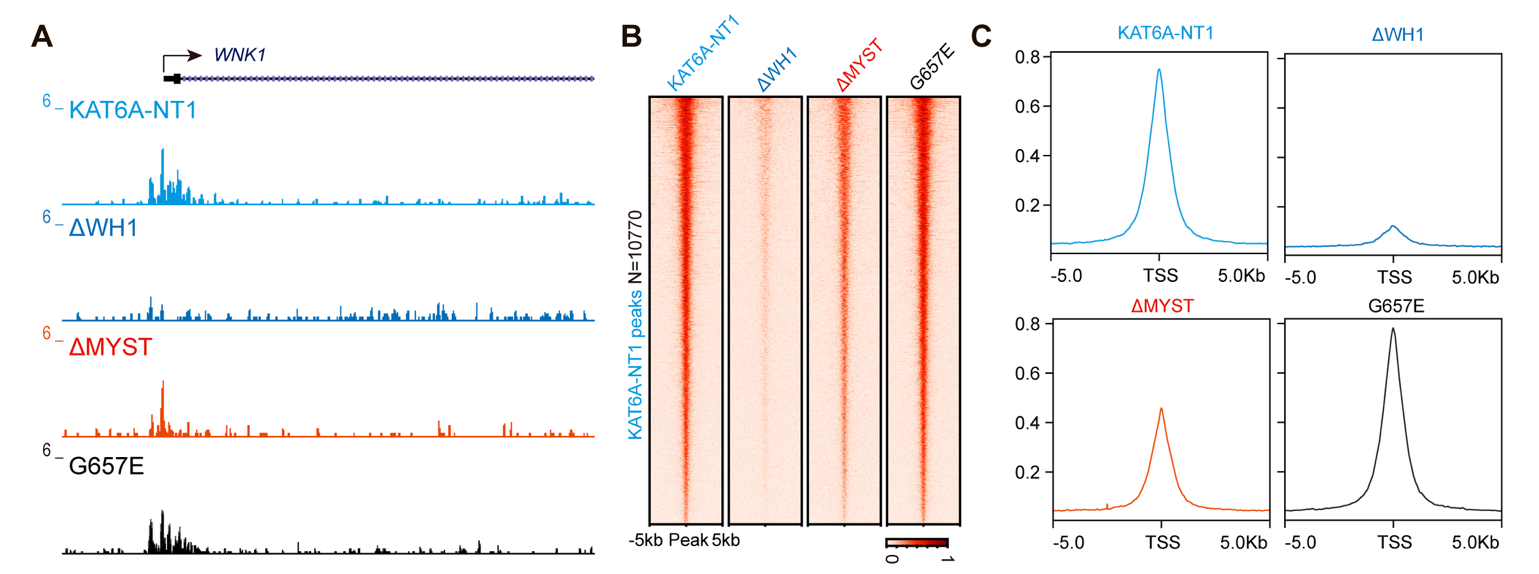


**Fig. S4. The MYST Domain Facilitates KAT6A Chromatin Occupancy.**

**A, B.** Genome browser track examples for KAT6A-NT1(1-780) and KAT6A-NT1 mutants in HEK293T cells. The deletion of the WH1 domain, responsible for CpG island binding, eliminates KAT6A occupancy, while deletion of the MYST domain—but not the acetyltransferase activity—reduces KAT6A occupancy, highlighting the MYST domain’s role in promoting chromatin binding.

**C.** Metaplot analyses of KAT6A-NT1-ΔMYST show reduced KAT6A occupancy across the genome compared to KAT6A-NT1 and the catalytically-dead KAT6A-NT1 mutant.

Fig. S5.


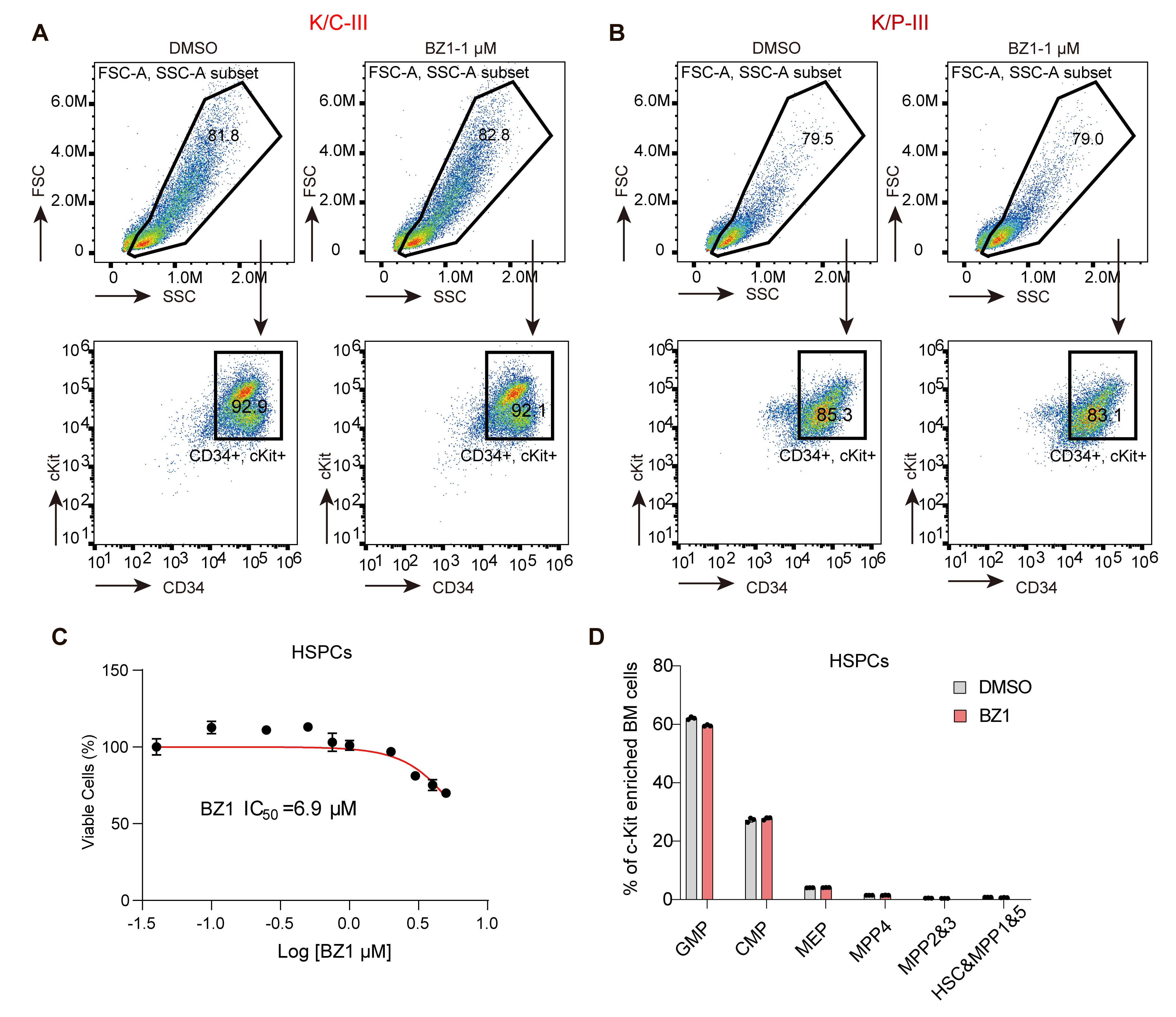
**Fig. S5. BZ1 exhibits minimal effects on K/C-III and K/P-III leukemia cell differentiation and preserves normal hematopoietic progenitor viability**

**A, B.** Flow cytometry analysis of leukemia cells treated with 1 µM BZ1 for 5 days.

**C.** Viability of c-Kit-enriched HSPCs following BZ1 (0–5 µM) treatments for 3 days (n=3).

**D.** Quantification of hematopoietic progenitor cell percentages in c-Kit-enriched HSPCs after 3-day BZ1 treatment (2 µM), measured by spectral flow cytometry.

Fig. S6.


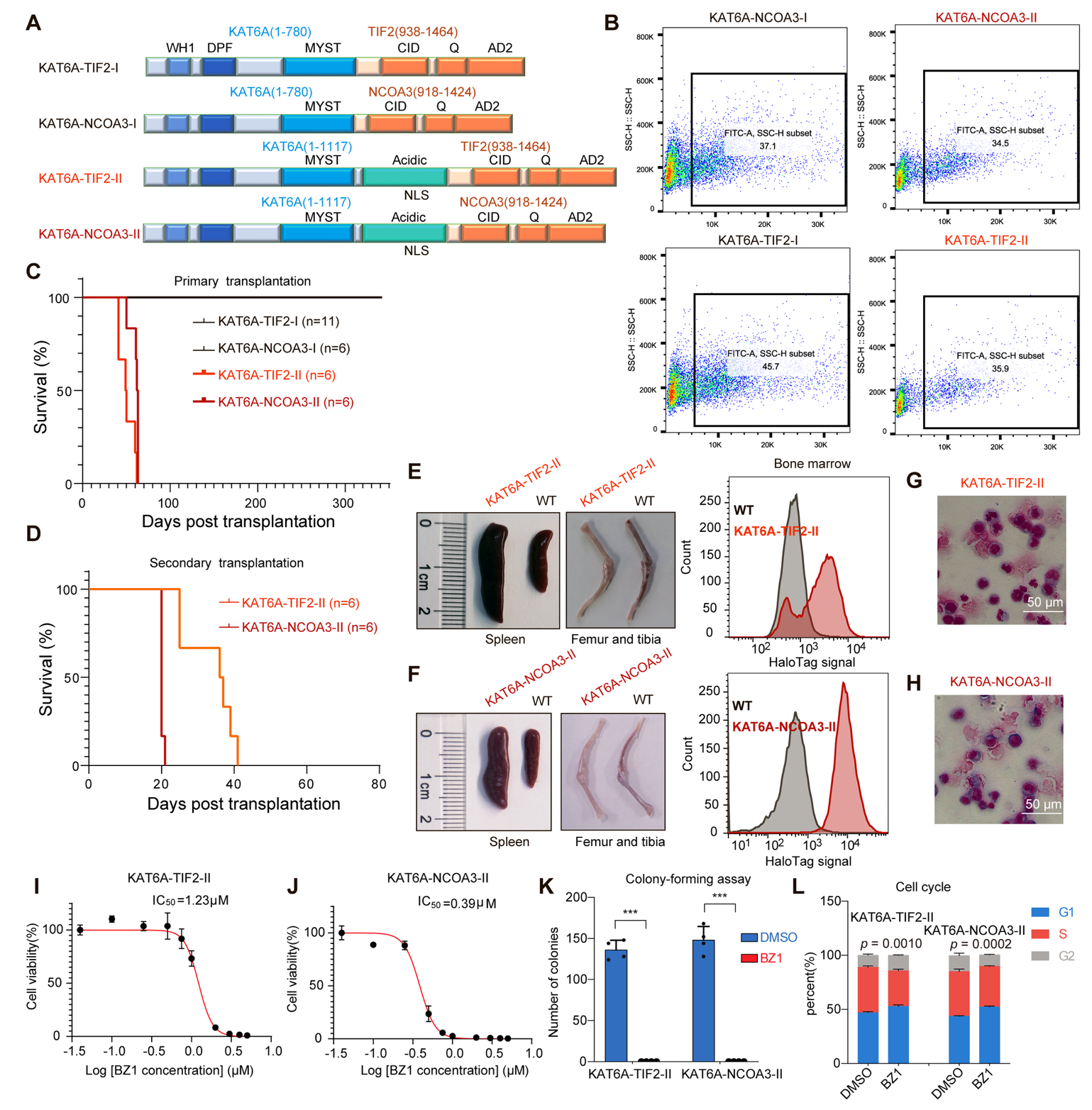


**Fig. S6. Generation of KAT6A-TIF2 and KAT6A-NCOA3 leukemia models.**

**A.** Schematic design of KAT6A-TIF2 and KAT6A-NCOA3 constructs for establishing leukemia models from murine HSPCs.

**B.** Transduction efficiency of KAT6A-TIF2 and KAT6A-NCOA3 in HSPCs, assessed via HaloTag R110 staining and flow cytometry analysis.

**C.** Kaplan-Meier survival curves for C57BL/6J mice transplanted with HSPCs transduced with KAT6A-TIF2 and KAT6A-NCOA3 constructs, demonstrating their oncogenic potential (n=6-11 mice per group). Both leukemia models depend on the acidic domain of KAT6A, likely due to a nuclear localization sequence within this domain that facilitates nuclear distribution.

**D.** Secondary transplantation of primary leukemic spleen cells from KAT6A-TIF2-II and KAT6A-NCOA3-II models (n=6 mice).

**E, F.** Endpoint analyses of leukemic mice bearing KAT6A-TIF2-II and KAT6A-NCOA3-II revealed pale bone marrow in the femur and tibia, along with splenomegaly due to leukemic blast infiltration.

**G, H.** Wright-Giemsa staining of leukemic cells from KAT6A-TIF2-II and KAT6A-NCOA3-II models revealed a mixture of mature and immature myeloid cells, many exhibiting monocytic features, alongside blast forms.

**I, J.** Dose-dependent inhibition of BZ1 on KAT6A-TIF2-II and KAT6A-NCOA3-II leukemia cells, with corresponding IC50 values indicated.

**K.** BZ1 treatment suppresses the colony-forming ability of KAT6A-TIF2-II and KAT6A-NCOA3-II leukemia cells.

**L.** BZ1 induces G1 cell cycle arrest in KAT6A-TIF2-II and KAT6A-NCOA3-II leukemia cells.

**Fig. S7**


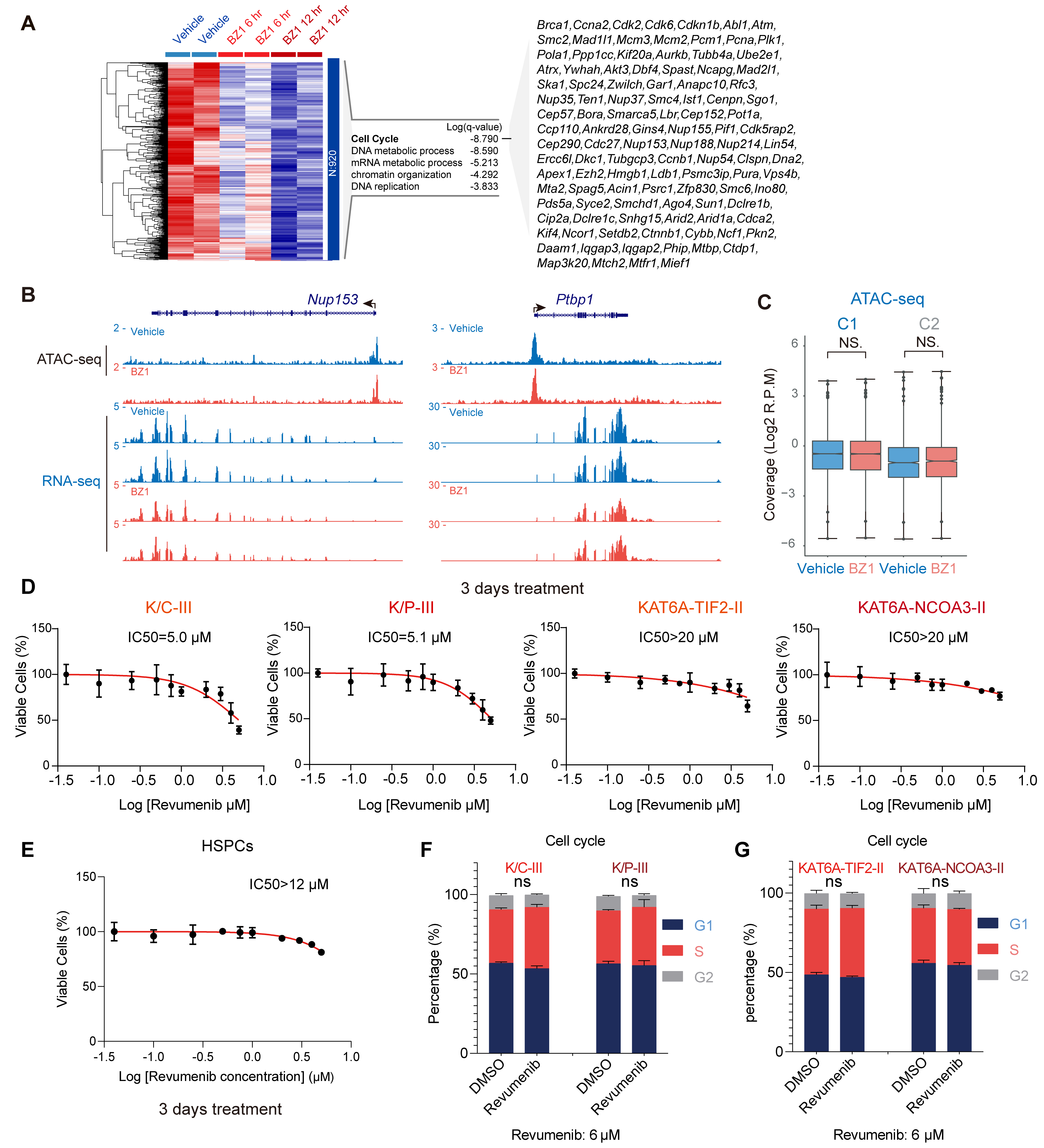


**Fig. S7. BZ1-Induced Transcriptional Changes and A485-Induced Differentiation**

**A.** Heatmap illustrating transcriptional downregulation in K/C-III leukemia cells following BZ1 treatment for 6 and 12 hours. Downregulated genes are enriched in pathways related to the cell cycle, DNA metabolic processes, chromatin organization, and DNA replication. Cell cycle-associated genes are highlighted on the right.

**B.** Tracks example of ATAC-seq and RNA-seq at *Nup153* and *Ptbp1* loci. K/C-III leukemia cells were treated with BZ1 for 6 hours before ATAC-seq.

**C.** Boxplot analysis of ATAC-seq signals for cluster 1 and 2 gene promoters.

**D, E.** KAT6A-fusion leukemic cells treated with Revumenib (0–5 μM, 3 days) showed limited reduction in viability (IC50 >5 μM), compared to HSPCs.

**F, G.** High-dose Revumenib (6 μM) treatment for 3 days failed to induce cell cycle arrest in KAT6A-fusion leukemic cells.

**Fig. S8**


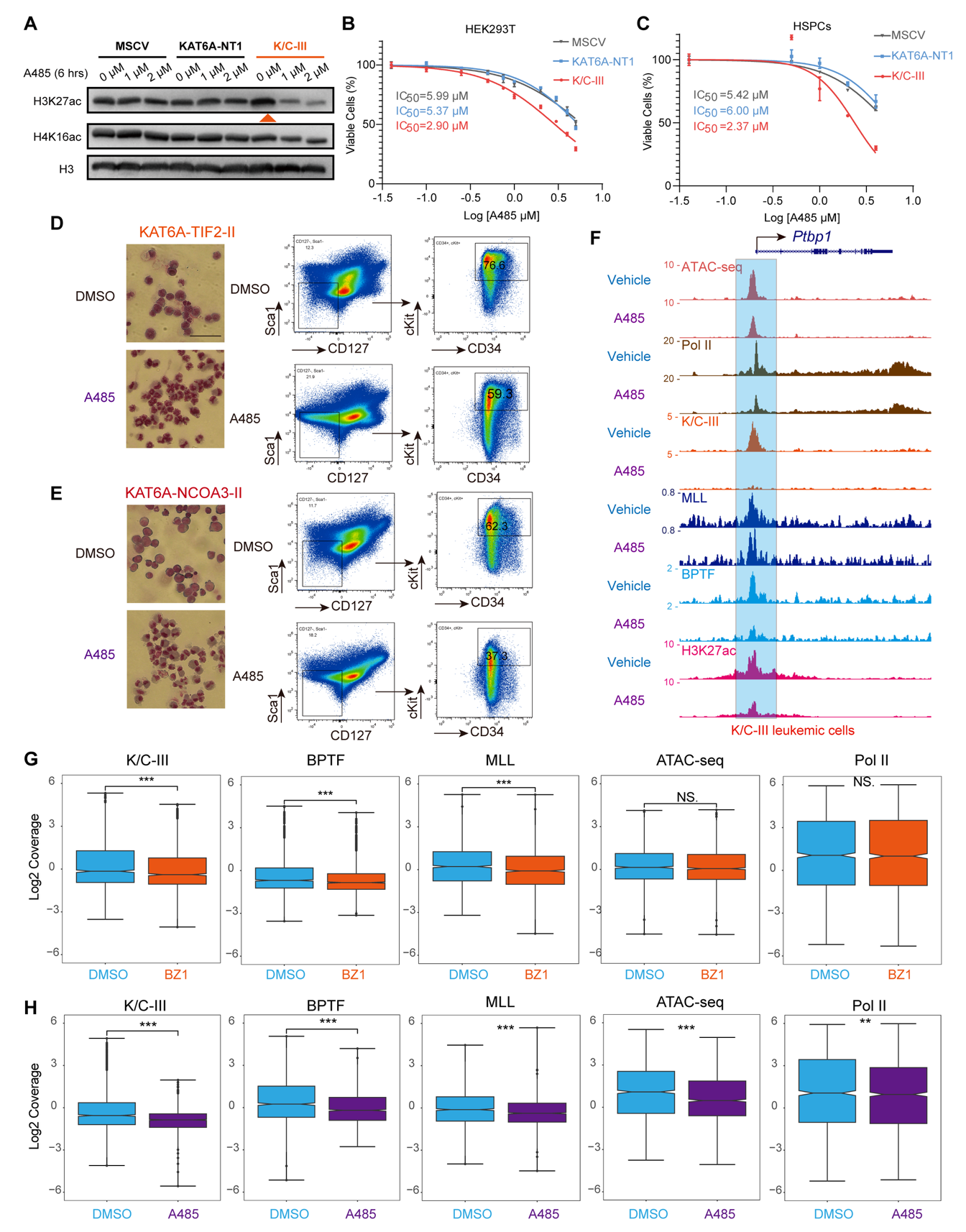


**Fig. S8. A485 treatment decreases the recruitment of K/C-NURF-MLL module.**

**A, B.** HEK293T cells expressing K/C-III exhibited elevated H3K27ac levels compared to KAT6A-NT1 or empty vector (MSCV) controls. Short-term A-485 treatment (6 hours) preferentially reduced H3K27ac and H4K16ac marks in K/C-III cells. Dose-response assays further revealed a 2-fold lower IC50 for A-485 in KAT6A-CBP cells (2.90 μM) compared to KAT6A-NT (5.37 μM) or MSCV controls (5.99 μM).

**C.** K/C-III-expressing HSPCs showed 2-fold greater sensitivity to A485 in viability assays.

**D, E.** A485 treatment for 7 days induces differentiation in KAT6A-TIF2 and KAT6A-NCOA3 leukemia cells.

F. Track examples of K/C-III, MLL, BPTF, Pol II and ATAC-seq signals at *Ptbp1* gene.

G, H. Boxplot analysis of K/C-III, BPTF, MLL, ATAC-seq, and Pol II signals at A485 downregulated genes.

**Fig. S9**


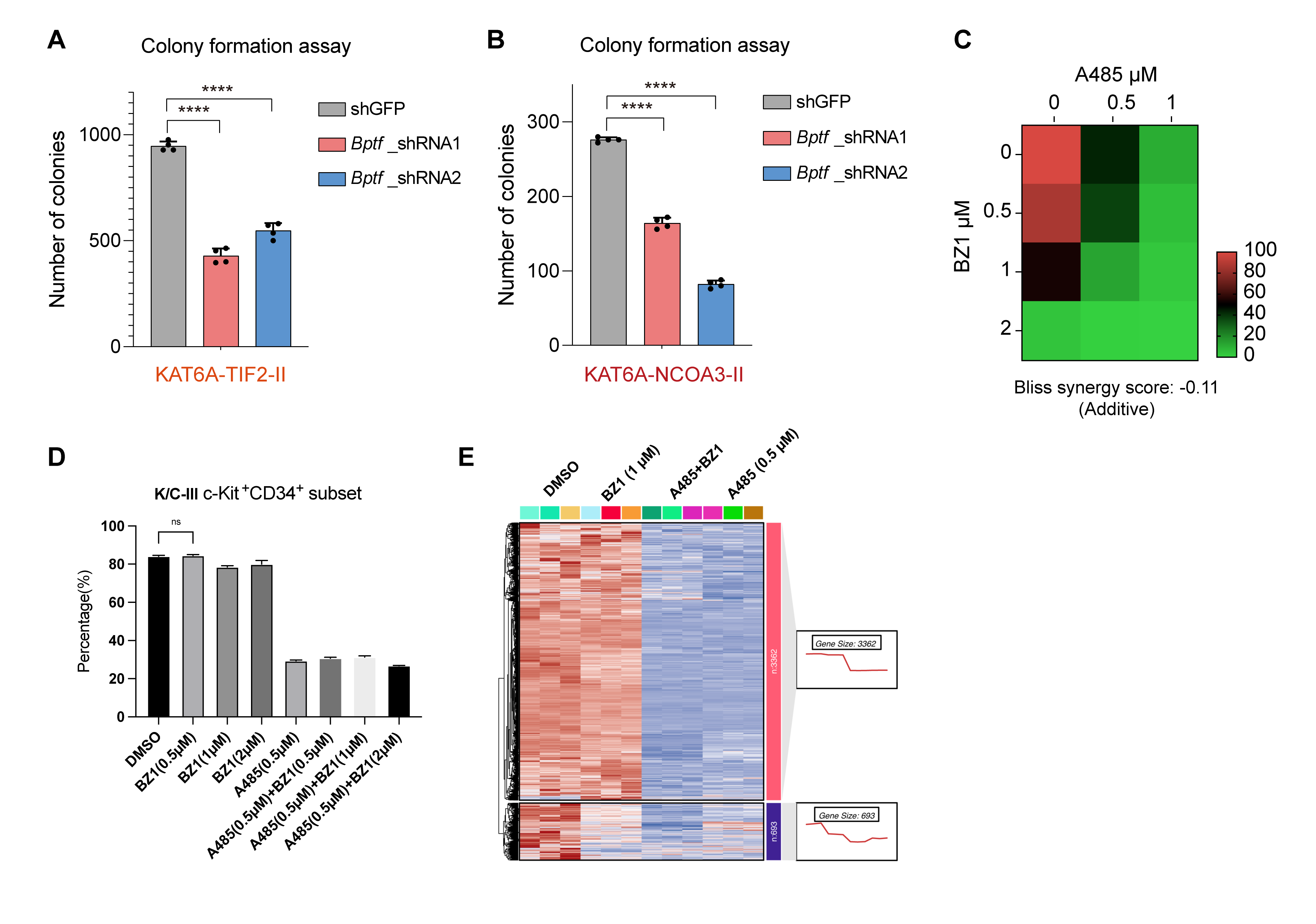


**Fig. S9. Additive effects of BZ1 and A485 in K/C-III leukemia inhibition.**

**A, B.** BPTF knockdown by shRNAs reduced colony formation in KAT6A-NCOA3A and KAT6A-TIF2 leukemic cells versus controls

**C.** BZ1 (0.5–2 μM) and A-485 (0.5–1 μM) Co-treatment in K/C-III leukemic cells yielded a Bliss synergy score of −0.11, indicating additive effects (score ≈ 0) rather than synergy. Bliss synergy score was calculated by SynergyFinder web version 3.0 (https://synergyfinder.fimm.fi).

**D.** BZ1 co-treatment did not further reduce the population of c-Kit+CD34+ leukemic cells compared to A485 treatments (Figure 6F), suggesting no enhanced differentiation effect.

**E.** RNA-seq of K/C-III leukemic cells treated with low-dose BZ1 (1 μM) and A485 (0.5 μM) revealed that co-treated cells shared most of the downregulated genes with A485-treated cells. No unique pathways or gene sets were suppressed in co-treated cells.

**Fig. S10**

**
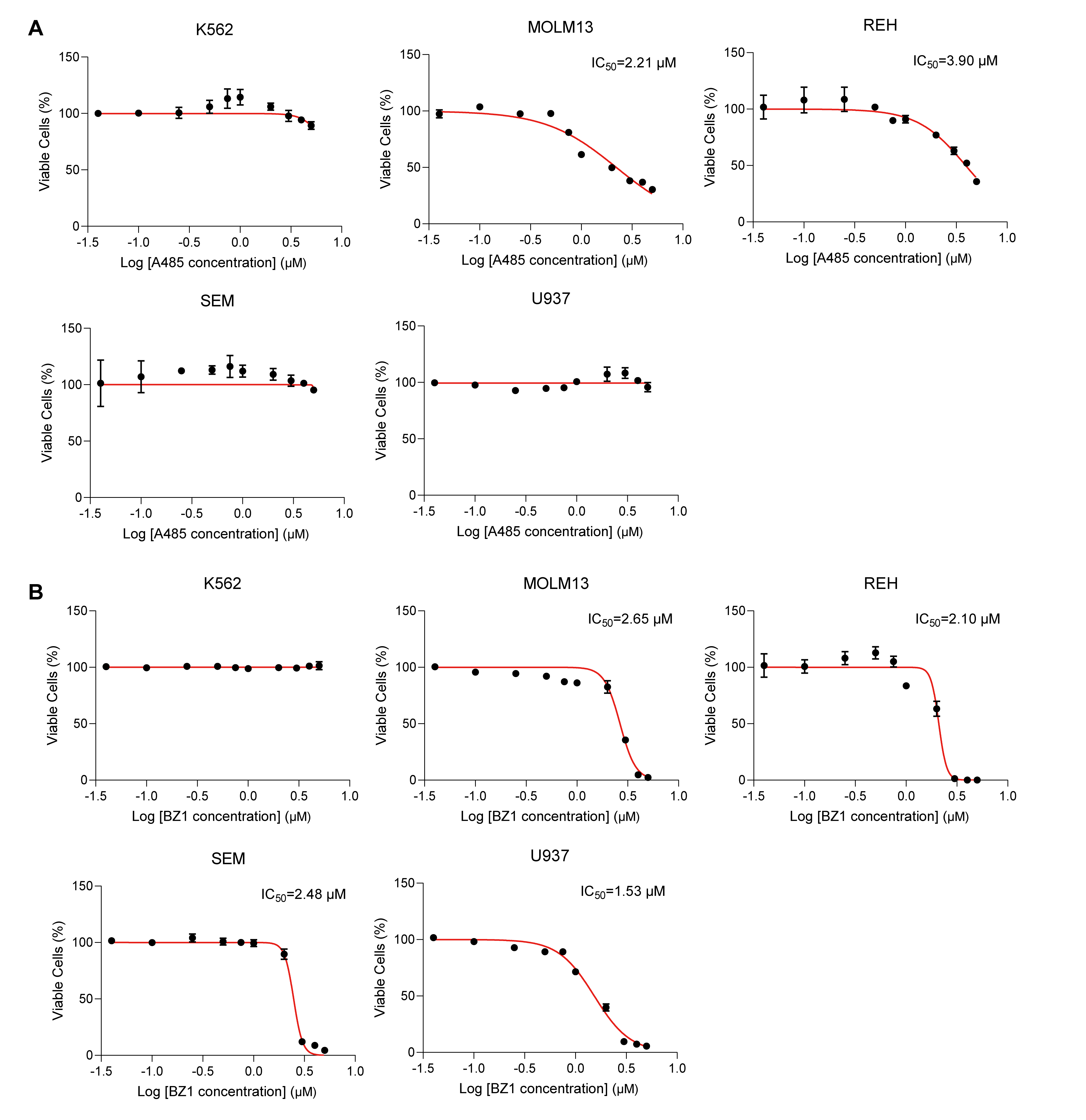
**

**Fig. S10. Cell proliferation assays of non-KAT6A human leukemia cell lines to A485 and BZ1 treatments**

**A.** Evaluation of the therapeutic potential of A-485 in common non-KAT6A human leukemia cell lines, including K562(BCR-ABL1^+^), MOLM13(MLL-AF9^+^), SEM (MLL-AF4^+^), U937, and REH. A485 exhibited minimal anti-proliferative effects in K562, SEM, and U937, with modest activity observed in MOLM13 and REH cells (IC50 = 2.21 μM and 3.90 μM, respectively).

**B.** Dose-response effects of BZ1 in K562, MOLM13, SEM, U937, and REH. BZ1 exhibited efficacy in these leukemia cell lines with IC50 from 1.5 μM to no response, which is large than the IC50 we obtained with KAT6A fusion leukemia cells (0.39-1.2 μM).
