# Supplementary figures and images for: KAT6A chimeras form a self-reinforcing epigenetic module with NURF and MLL/COMPASS to sustain AML
Source: Genome Biol. 2025 Aug 19;26:253. doi: 10.1186/s13059-025-03743-y (PMC12366150; doi:10.1186/s13059-025-03743-y)

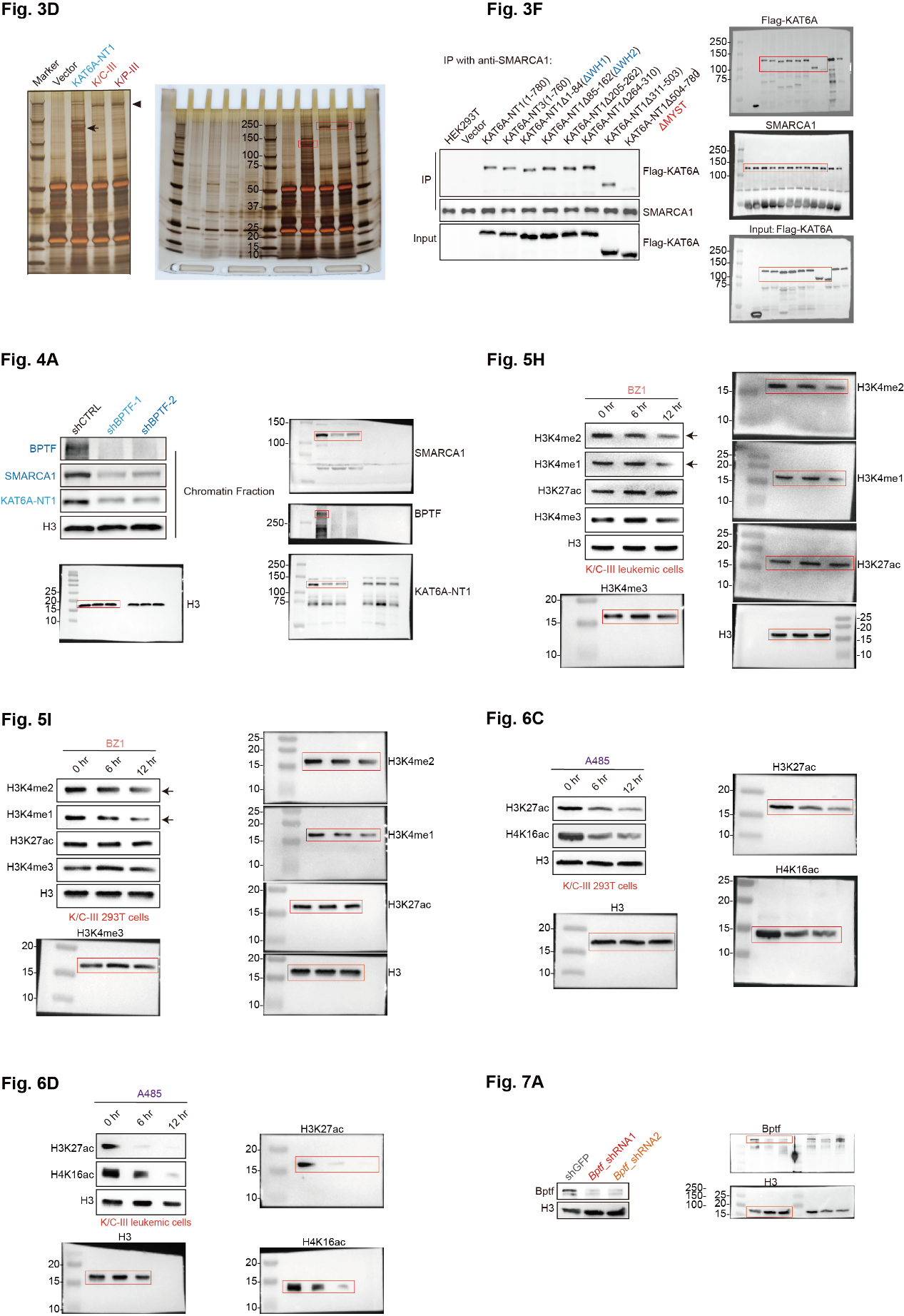

Supplement: Supplementary file 3 — Additional file 3: Uncropped western blot and gel images [file 13059_2025_3743_MOESM3_ESM.docx]
